# Risk and protective factors for suicidal ideation and suicide attempts among Chinese university students: a systematic review and meta-analysis of longitudinal studies

**DOI:** 10.1186/s12889-026-27430-0

**Published:** 2026-04-20

**Authors:** Xuhong Song, Xiaomeng Wang, Pan Jiang, Yucheng Zhong, Zhichen Guo, Qin Wang, Yan Cai, Dongbo Tu

**Affiliations:** 1 School of Psychology, Jiangxi Normal University, Nanchang, Jiangxi China; 2 Student Affairs Office, Jiangxi Vocational College of Foreign Studies, Nanchang, China; 3 Wenxin Academy, Henan Open University, Zhengzhou, China; 4 School of Early Childhood Education, Shangrao Preschool Education College, Shangrao, China; 5 School of Management and Economics, The Chinese University of Hong Kong, Shenzhen, China; 6 Jiangxi Laboratory of Philosophy and Social Sciences (Data Science and Intelligent Psychological Assessment and Service Laboratory), Jiangxi Normal University, Nanchang, China

**Keywords:** Suicidal ideation, Suicide attempts, Risk factors, Protective factors, Systematic review and meta-analysis, Chinese university students

## Abstract

**Background:**

Suicide among university students in China constitutes a critical public health challenge. Although numerous studies have examined risk factors, most have relied on cross-sectional designs. Evidence from longitudinal studies, particularly regarding protective factors, remains limited. This systematic review and meta-analysis synthesizes longitudinal evidence to identify risk and protective factors for suicidal ideation and suicide attempts among Chinese university students.

**Methods:**

Six electronic databases (three English-language and three Chinese-language databases) were systematically searched for longitudinal studies published up to October 2024. Eligible studies investigated predictors of suicidal ideation or suicide attempts among Chinese university students. Random-effects meta-analyses were conducted to estimate pooled effect sizes, expressed as odds ratios (ORs). Meta-regression analyses were used to examine temporal moderators, and heterogeneity and publication bias were assessed.

**Results:**

Twenty-two longitudinal studies comprising 953,651 participants were included. The strongest predictors of subsequent suicidal ideation were prior suicidal ideation, anxiety, and early morning awakening. Suicide attempts were most strongly associated with prior non-suicidal self-injury, insomnia symptoms, and academic pressure. Higher levels of social support were associated with a reduced risk of suicidal ideation, whereas other examined protective factors were not statistically significant. Meta-regression analyses indicated that the predictive strength of depressive symptoms for suicidal ideation attenuated with longer follow-up periods.

**Conclusions:**

Sleep disturbances, academic pressure, and prior suicidal ideation may represent important targets for early identification among Chinese university students. The findings highlight the importance of addressing both psychological vulnerabilities and contextual stressors in suicide prevention efforts. Future longitudinal research should adopt standardized measurements to enhance comparability and inform culturally tailored population-level prevention strategies.

**Supplementary Information:**

The online version contains supplementary material available at 10.1186/s12889-026-27430-0.

## Introduction

 Suicide represents a major global public health concern, with profound consequences for individuals, families, and communities. Worldwide, nearly 700,000 people die by suicide each year, a mortality burden that exceeds several major infectious diseases. Suicide is also the third leading cause of death among individuals aged 15–29 years, highlighting its disproportionate impact on young people [1]. Within this global context, university students constitute a particularly vulnerable population. The transition to higher education exposes students to multiple stressors, including lifestyle changes, academic demands, socioeconomic pressures, and uncertainty about future career prospects. These challenges have been associated with elevated levels of psychological distress and mental health problems among university students [2–4]. Although university life promotes intellectual development and personal growth, it may simultaneously exacerbate mental health difficulties and, in some cases, contribute to suicidality. Competitive academic environments, social isolation, and reduced familial support may further heighten psychological vulnerability during this developmental period.

The burden of suicide is also a major concern in China. Although systematic national statistics on deaths among university students are not routinely publicly available, available regional evidence—although limited in recency—indicates that suicide constitutes a substantial proportion of student deaths. For example, a provincial investigation reported that suicide accounted for 37.7% of all deaths among university students between 2002 and 2006. Within the category of unnatural deaths, suicide accounted for 47.2% of cases among university students [5]. Although national data suggest that overall suicide rates in China have declined over the past two decades, suicide risk among university students remains a significant concern [5]. A meta-analysis estimated pooled prevalence rates of 10.8% for suicidal ideation and 2.7% for suicide attempts among Chinese university students between 2010 and 2020 [6]. Moreover, recent evidence suggests that the decline in suicide rates may have slowed in recent years [7]. These patterns indicate that existing prevention efforts may not fully address the contextual factors shaping suicidality among Chinese university students. Identifying risk and protective factors within this sociocultural environment is therefore essential for developing more effective and context-sensitive prevention strategies. Insights from the Chinese sociocultural context may also contribute to the broader suicide literature by highlighting pathways potentially relevant to other collectivist or non-Western societies. Previous research has described the Chinese sociocultural context as often characterized by collectivist cultural values, strong family expectations, and high levels of academic competition [8–10].

A growing body of research has examined psychological, behavioral, and social predictors of suicidality among university students. Factors such as depression, anxiety, hopelessness, impulsivity, adverse life events, and sleep disturbances have frequently been associated with suicidal behaviors [11, 12]. However, findings remain inconsistent across studies. While some studies demonstrate that psychological distress and impaired stress adaptation mechanisms robustly predict suicidal thoughts [13]. others report weaker or non-significant associations between certain life stressors and suicidal ideation [14]. Inconsistencies are particularly evident in demographic and behavioral predictors. For example, evidence regarding gender differences remains mixed, with some studies reporting higher risk among female students and others finding no significant gender-based differences [15]. Similarly, family-related factors—including parenting styles, family conflict, and family history of suicide—have shown variable associations across studies [16]. Behavioral factors such as substance use, disordered eating patterns, and sleep disturbances also demonstrate heterogeneous findings across investigations [12, 17]. Collectively, these findings suggest that suicidality among university students reflects a complex interaction of multiple risk factors rather than a single causal pathway. In addition to psychosocial and behavioral determinants, emerging evidence from other domains, including pharmacological research, has also explored potential associations with suicidal behavior, although findings remain inconclusive and context-specific [18].

In addition to risk factors, protective factors are increasingly recognized as important components of suicide prevention research. Protective resources may buffer the effects of stressors or influence individuals’ responses to adversity [19]. Empirical studies have identified factors such as self-esteem, optimism, resilience, hope, self-compassion, and social support as potential protective influences [20, 21]. Qualitative research has also highlighted the role of close interpersonal ties, higher levels of well-being, and engagement in religious or spiritual practices as protective factors [22]. However, the relevance of these factors may differ across cultures. Whereas autonomy and self-efficacy are often emphasized in Western societies, collectivistic contexts such as China may place greater importance on family cohesion, academic belongingness, and integration into peer networks. These differences highlight the importance of examining both risk and protective factors within specific sociocultural contexts.

Despite a growing body of research, several important gaps remain in the literature. First, existing systematic reviews have often focused on prevalence estimates or specific student subgroups rather than providing a comprehensive synthesis of risk and protective factors among Chinese university students [16, 23]. Second, many previous meta-analyses have relied primarily on cross-sectional studies. Such designs limit the ability to establish temporal relationships between predictors and suicidal outcomes, making it difficult to distinguish correlates from prospective risk factors. Third, much of the available longitudinal evidence originates from Western contexts. In these settings, suicidal behaviors are often conceptualized primarily in terms of individual psychological distress [24, 25]. In contrast, suicidality among Chinese university students may also be shaped by broader social and cultural factors, including family expectations, academic pressure, and stigma surrounding mental health [26, 27]. Finally, existing findings remain heterogeneous due to variations in study design, measurement approaches, and cultural contexts. Synthesizing longitudinal evidence is therefore necessary to clarify which factors demonstrate consistent predictive value.

To address these gaps, the present study conducts a systematic review and meta-analysis of longitudinal studies examining predictors of suicidal ideation and suicide attempts among Chinese university students. By synthesizing prospective evidence from both English- and Chinese-language studies, this review aims to identify temporally robust risk and protective factors, examine heterogeneity in effect sizes, and highlight areas requiring further research. By integrating longitudinal evidence with attention to sociocultural context, this study seeks to advance understanding of suicidality among Chinese university students and inform the development of more targeted and contextually relevant suicide prevention strategies in higher education settings.

## Methods

### Protocol registration and reporting standards

The protocol for this systematic review and meta-analysis was prospectively registered in PROSPERO (CRD420251040151). The study was conducted and reported in accordance with the Preferred Reporting Items for Systematic Reviews and Meta-Analyses (PRISMA) 2020 guidelines (Supplementary material 1).

### Search strategy

**Fig. 1:**
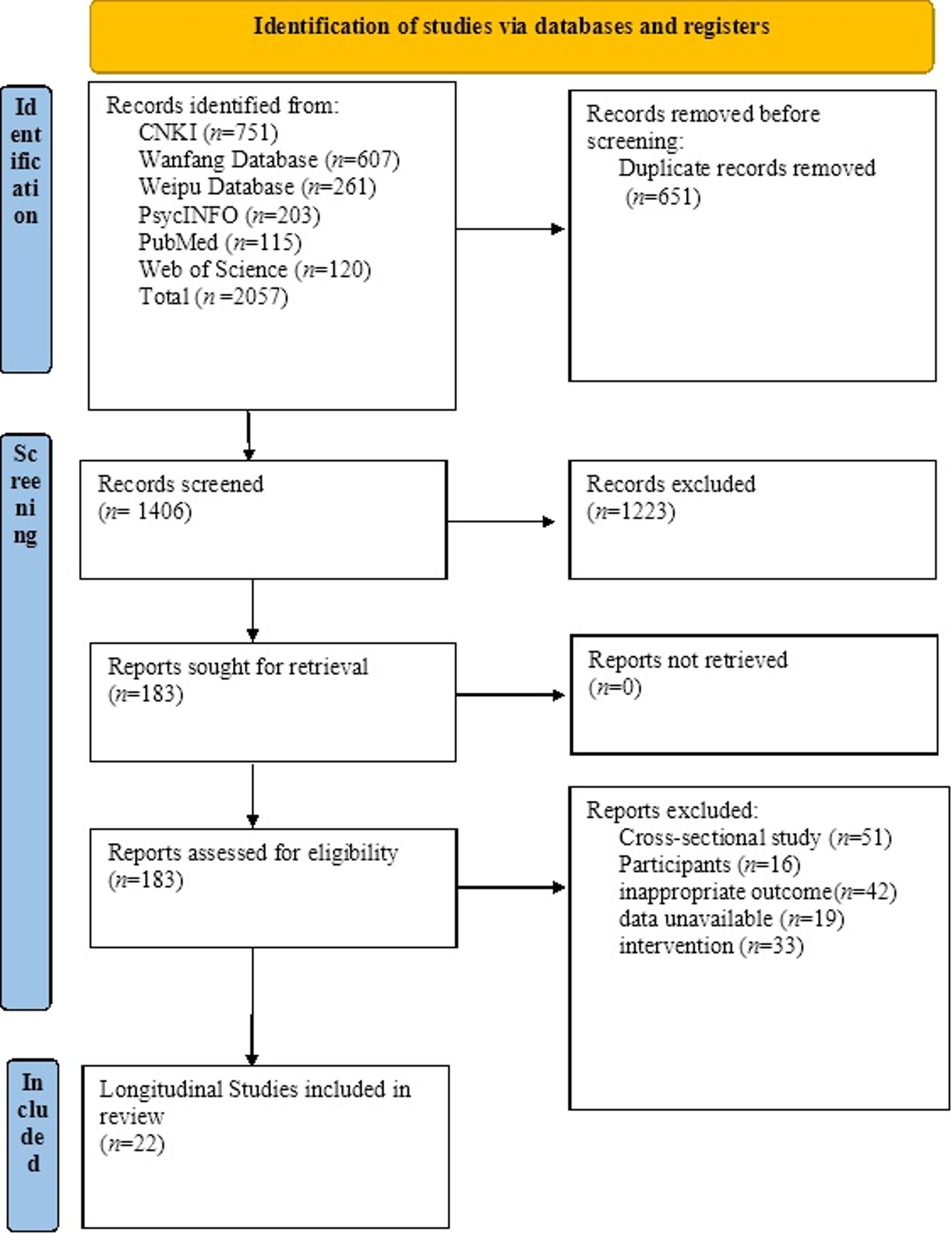
Flowchart of study identification and selection

We systematically searched three major Chinese academic databases (China National Knowledge Infrastructure [CNKI], Wanfang, and Weipu) and three international databases (PubMed, PsycINFO, and Web of Science) from database inception to October 31, 2024. Searches were restricted to publications in Chinese or English, and only peer-reviewed journal articles were considered eligible. The search strategy combined Boolean operators across three key concept clusters: (1) population terms (“China” OR “Chinese” AND “university student*” OR “undergraduate” OR “freshman”); (2) suicide-related outcomes (“suicid*,” “suicidal ideation,” “suicide attempt*,” “suicide plan”); and (3) study design filters (“longitudinal,” “prospective,” “cohort”). Full search strategies for all databases are provided in Supplementary material 2. To enhance comprehensiveness, reference lists of all included articles were manually screened to identify additional eligible studies. The study selection process strictly followed PRISMA 2020 guidelines and is illustrated in Fig. 1.

### Inclusion and exclusion criteria

Studies were included if they met the following criteria: (a) were peer-reviewed original research articles written in Chinese or English; (b) examined undergraduate students enrolled in universities in mainland China, including comprehensive universities, vocational universities, and polytechnic institutions; (c) reported quantitative measures of suicidal ideation or suicide attempts using validated instruments or clearly defined survey items; and (d) employed prospective longitudinal or cohort study designs. Studies focusing on Chinese international students studying abroad or other overseas Chinese populations were not included, as their sociocultural environments may differ substantially from those of students studying in mainland China. When multiple articles were derived from the same dataset, only the most comprehensive or methodologically detailed report was retained to avoid duplication.

Studies were excluded if they met any of the following criteria: (a) did not operationalize suicidal ideation or suicide attempts as measurable outcomes; (b) used cross-sectional or qualitative designs that could not establish temporal relationships between predictors and outcomes; or (c) focused on clinical psychiatric samples (e.g., participants recruited from psychiatric hospitals or with diagnosed mental disorders).

### Study selection procedure

All identified records were imported into EndNote X9 (Clarivate Analytics) for reference management. Duplicates were removed prior to screening. In the first stage, three reviewers independently screened titles and abstracts against predefined eligibility criteria. Potentially relevant articles were then subjected to full-text review using the same standardized protocol, with detailed documentation of exclusion reasons. To identify additional studies, three reviewers manually screened the reference lists of included studies and relevant reviews. Only studies meeting the predefined eligibility criteria were retained. Discrepancies at any stage of the screening process were resolved through discussion until consensus was reached.

### Data extraction

A structured data extraction framework was applied, covering three domains: (1) study characteristics, including research design, methodological details, and sample features; (2) quantitative outcomes and statistical parameters, with particular attention to odds ratios (ORs) and their 95% confidence intervals; and (3) risk factors reported in each study. Two trained coders independently extracted data using a coding manual that was pilot-tested prior to formal data extraction to ensure clarity and consistency. Inter-rater reliability was assessed (Cohen’s κ = 0.82), indicating excellent agreement. Any discrepancies were addressed through re-examination of the original reports and, when necessary, resolved through discussion or consultation with a third reviewer. To ensure temporal precedence consistent with prospective study designs, risk factor measurements were restricted to baseline assessments when available.

### Study quality assessment

Study quality was assessed using the Newcastle–Ottawa Scale (NOS) (Stang, 2010), which evaluates cohort studies across three domains: selection of study groups (four items), comparability (one item), and outcome assessment (three items). The total NOS score ranges from 0 to 9. To minimize potential subjectivity in quality assessment, two reviewers independently evaluated each study using pre-specified NOS criteria. Any discrepancies in scoring were resolved through discussion, and when necessary, consultation with a third reviewer. This standardized assessment process helped to enhance consistency and reduce potential subjective bias. Consistent with prior meta-analyses, studies scoring ≥ 6 were considered to be of relatively high methodological quality and were retained for analysis.

### Data analysis

We employed standard meta-analytic methods to quantify associations between identified risk and protective factors and suicide-related outcomes. Because all included studies were longitudinal in design, temporal relationships could be examined, strengthening the interpretation of temporal associations between predictors and suicidal ideation and suicide attempts. Effect sizes were extracted or computed systematically, with adjusted odds ratios (ORs) prioritized when available. As individual studies often examined multiple predictors, each could contribute more than one effect size. To ensure consistency across studies, we used data from the final follow-up assessment.

To address heterogeneity in both predictors and outcomes, we applied a structured classification approach. Effect sizes were first grouped by outcome type (suicidal ideation or suicide attempts) and then further classified according to specific categories of risk and protective factors. Meta-analyses were performed only when at least two effect sizes were available for the same predictor–outcome pairing. Because the operational definitions and measurement instruments for suicidal ideation and suicide attempts varied across studies (e.g., standardized psychological scales or structured survey items), outcomes were harmonized based on the conceptual definitions reported in the original studies. Specifically, suicidal ideation was defined to include a range of self-reported thoughts about suicide, from passive death wishes to more active ideation or planning, whereas suicide attempts were defined as self-reported engagement in potentially self-injurious behaviors with at least some intent to die. To ensure comparability across studies, outcomes were grouped based on whether they reflected suicidal thoughts or enacted self-harm behaviors. Although this approach does not distinguish between different levels of severity (e.g., passive versus active ideation), it allows for a conceptually consistent synthesis of longitudinal evidence across studies with heterogeneous measurement approaches. Studies were included only if suicidal ideation or suicide attempts were clearly operationalized as measurable outcomes.

Random-effects models with restricted maximum likelihood (REML) estimation were used, and Knapp–Hartung corrections were implemented to minimize small-sample bias. Pooled ORs with 95% confidence intervals (CIs) were calculated using inverse-variance weighting. When multiple effect sizes were extracted from the same study, robust variance estimation was used to account for within-study dependence. We evaluated between-study heterogeneity using Cochran’s Q test and the I² statistic. I² values greater than 50% were interpreted as indicating substantial heterogeneity [28].

Publication bias was assessed through two complementary methods: visual inspection of funnel plot asymmetry and Egger’s regression test. To explore sources of heterogeneity, univariate random-effects meta-regression analyses were conducted when substantial heterogeneity was detected (I²≥50%). Because meta-regression requires sufficient statistical power, analyses were performed only when at least ten effect sizes were available for a given predictor. Each model tested a single covariate (e.g., sex proportion, residence, prior suicide attempts, or prevalence of depressive symptoms), with Knapp-Hartung correction applied. Continuous covariates were mean-centered, and categorical covariates were dummy-coded. Effect sizes were interpreted as follows: an OR of 1.0 (95% CI includes 1.0) indicated no significant association; an OR greater than 1.0 (95% CI lower bound > 1.0) indicated a potential risk factor; and an OR less than 1.0 (95% CI upper bound < 1.0) indicated a potential protective factor. All analyses were conducted in R version 4.2.2 using the metafor package.

## Results

### Study characteristics

A total of 2,057 records were identified through the database searches. After title and abstract screening, 1,874 records were excluded because they did not meet the eligibility criteria regarding study design or outcome measures. Full-text articles of 183 potentially eligible studies were assessed, of which 22 longitudinal studies involving 953,651 participants were ultimately included in the meta-analysis. The study selection process is presented in the PRISMA flow diagram (Fig. 1), and the key characteristics of the included studies are summarized in Supplementary material 3.

The included studies were published between 2005 and 2024. Sample sizes ranged from 197 to 67,905 participants, and follow-up durations varied from 1 to 48 months. In cases where multiple publications were derived from overlapping datasets but reported different predictors, the relevant effect estimates were retained and analyzed for the corresponding predictors. Statistical dependence among effect sizes originating from the same dataset was addressed using robust variance estimation, as described in the Methods section. According to the Newcastle–Ottawa Scale, all included studies were of relatively high methodological quality. Detailed quality assessment results are presented in Supplementary material 4.

### Risk factors for suicidal ideation and suicide attempts

Across the 22 longitudinal studies, the meta-analysis identified 26 predictors examined for suicidal ideation and 8 predictors for suicide attempts, of which several reached statistical significance. Following Franklin et al. [19], predictors were categorized into five domains: demographic factors, behavioral and health-related factors, individual psychological factors, interpersonal factors, and sociocultural context. Complete results for all predictors are presented in Tables 1 and 2.

#### Suicidal ideation

No demographic factors were significantly associated with suicidal ideation; specifically, female sex and urban residence were not significant predictors.

In contrast, several individual psychological factors showed significant associations, with pooled odds ratios ranging from 1.87 to 3.38. The strongest predictors included prior suicidal ideation (OR = 3.38, 95% CI [1.23–9.29]), anxiety (OR = 2.27, 95% CI [2.13–2.41]), prior non-suicidal self-injury (NSSI; OR = 2.18, 95% CI [1.77–2.69]), history of mental illness (OR = 1.94, 95% CI [1.22–3.07]), and depressive symptoms (OR = 1.87, 95% CI [1.47–2.38]). Within this domain, prior suicide attempts did not show a statistically significant association (OR = 2.51, 95% CI [0.76–8.31]), and suboptimal health status also did not reach statistical significance (OR = 2.04, 95% CI [1.00–4.17]), although the estimate was borderline.

Among behavioral and health-related factors, early morning awakening (OR = 2.39, 95% CI [1.65–3.46]), insomnia symptoms (OR = 1.73, 95% CI [1.40–2.13]), and alcohol use (OR = 1.65, 95% CI [1.20–2.26]) were significantly associated with suicidal ideation, whereas somatic pathological symptoms were not (OR = 1.36, 95% CI [0.89–2.07]).

Within the interpersonal factors, poor family functioning showed a modest but borderline statistically significant association with suicidal ideation (OR = 1.85, 95% CI [1.00–3.41]). In the sociocultural domain, negative life events (OR = 1.65, 95% CI [1.28–2.11]), academic pressure (OR = 1.54, 95% CI [1.21–1.95]), and stress (OR = 1.45, 95% CI [1.09–1.92]) were significantly associated with suicidal ideation. In contrast, parental marital status (OR = 1.40, 95% CI [0.99–1.99]) and childhood emotional abuse (OR = 1.14, 95% CI [0.84–1.54]) did not show statistically significant associations. Detailed estimates are shown in Table 1. Forest plots for suicidal ideation predictors are provided in Supplementary material 5.

**Table 1:** Risk and protective factors for suicidal ideation

Domain	Predictors	k	*N*	Odds Ratio	95%CI	*p*-value	I²	Egger’s *p*
**Demographic factors**	Residence(urban)	7	51,775	1.09	[0.76,1.56]	0.645	87.21%	0.76
	*Sex (female)*	9	57,930	0.99	[0.89,1.11]	0.890	50.82%	0.63
	*Residence (Town)*	4	10,887	0.98	[0.69,1.40]	0.920	64.16%	0.94
**Behavioral & Health-Related** **Factors**	Early morning awakening	3	136,143	2.39	[1.65,3.46]	< 0.001	77.68%	0.77
	Insomnia symptoms	5	154,308	1.73	[1.40,2.13]	< 0.001	90.35%	0.23
	Alcohol use	2	5535	1.65	[1.20,2.26]	< 0.01	0%	NA
	Sleep duration	4	204,048	1.61	[0.99,2.63]	0.054	96.34%	0.32
	Somatic pathological symptoms	4	43,346	1.36	[0.89,2.07]	0.157	81.58%	0.48
**Individual Psychological** **Factors**	Prior suicide ideation	4	20,347	3.38	[1.23,9.29]	< 0.05	75.54%	0.09
	Prior suicide attempt	3	6896	2.51	[0.76,8.31]	0.131	79.38%	0.48
	Anxiety	2	67,905	2.27	[2.13,2.41]	< 0.001	0%	NA
	Prior NSSI	3	11,165	2.18	[1.77,2.69]	< 0.001	0%	0.86
	Suboptimal Health Status	3	1658	2.04	[1.00,4.17]	0.050	0%	0.12
	History of mental illness	5	44,256	1.94	[1.22,3.07]	< 0.01	42.73%	0.44
	Depressive symptoms	11	128,526	1.87	[1.47,2.38]	< 0.001	81.98%	0.72
	*Resilience*	2	5236	0.79	[0.57,1.09]	0.151	98.00%	NA
**Interpersonal Factors**	Family function Poor	2	42,814	1.85	[1.00,3.41]	< 0.05	70.29%	NA
	Family function Fair	2	42,814	1.59	[0.93,2.72]	0.091	78.67%	NA
	Parental marital status	3	5515	1.40	[0.99,1.99]	0.058	0%	0.12
	Childhood emotional abuse	2	4781	1.14	[0.84,1.54]	0.404	88.84%	NA
	*Social support: high*	4	72,153	0.66	[0.48,0.91]	< 0.01	98.76%	0.25
**Sociocultural Context**	Negative life events	3	15,270	1.65	[1.28,2.11]	< 0.001	0%	0.36
	Academic pressure	2	5118	1.54	[1.21,1.95]	< 0.001	0%	NA
	Stress	2	1558	1.45	[1.09,1.92]	< 0.01	0%	NA
	Single child status	4	37,571	1.10	[0.99,1.22]	0.088	0%	0.22
	*Family economic status: high*	5	10,120	0.92	[0.78,1.08]	0.288	12.70%	0.16

*k*=Number of independent samples analyzed; *N*= total number of participants in the analysis; *I² =* heterogeneity statistic; 95%CI = 95% confidence interval of pooled OR; *p* = p-value (two-tailed); *Q-test p-value*: p-value for Cochran’s Q test of heterogeneity. *Egger’s p*: p-value for Egger’s regression test of publication bias. *p < 0.05; **p < 0.01; ***p < 0.001. The analysis used a random effects model

#### Suicide attempts

For suicide attempts, three predictors showed statistically significant associations: prior NSSI (OR = 2.07, 95% CI [1.65–2.59]), academic pressure (OR = 1.94, 95% CI [1.39–2.72]), and insomnia symptoms (OR = 1.60, 95% CI [1.41–1.81]). Other examined factors, including female sex, prior suicidal ideation, prior suicide attempts, depressive symptoms, and history of mental illness, were not significantly associated with suicide attempts. Forest plots for predictors of suicide attempts are presented in Supplementary material 6. Corresponding results are summarized in Table 2.

**Table 2:** Risk and protective factors for suicide attempts

Domain	Predictors	k	*N*	Odds Ratio	95%CI	*p*-value	I²	Egger’s *p*
Demographic factors	female	2	10,758	0.91	[0.67,1.24]	0.552	2.82%	NA
Individual Psychological Factors	Prior NSSI	3	11,165	2.07	[1.65,2.59]	< 0.001	0%	0.299
	Prior suicide ideation	4	20,347	1.92	[0.85,4.38]	0.119	37.49%	0.194
	Prior suicide attempts	4	20,347	1.87	[0.76,4.61]	0.173	38.32%	0.330
	Depressive symptoms	2	7705	1.77	[0.95,3.29]	0.072	91.21%	NA
	History of mental illness	3	9211	1.57	[0.66,3.76]	0.311	45.55%	0.597
Sociocultural Context	academic pressure	2	5118	1.94	[1.39,2.72]	< 0.001	24.06%	NA
Behavioral Health-Related FactorsBehavioral Health-Related Factors	Insomnia symptoms	3	18,498	1.60	[1.41,1.81]	< 0.001	0%	0.608

*k*= Number of independent samples analyzed; *N* = total number of participants in the analysis; *I² =* heterogeneity statistic; 95%CI = 95% confidence interval of pooled OR; *p* = p-value (two-tailed); *Egger’s p*: p-value for Egger’s regression test of publication bias. *p < 0.05; **p < 0.01; ***p < 0.001. The analysis used a random effects model

### Protective factors for suicidal ideation and suicide attempts

High levels of social support were significantly associated with a reduced risk of suicidal ideation (OR = 0.66, 95% CI [0.48–0.91]). Other proposed protective factors, including resilience (OR = 0.79, 95% CI [0.57–1.09]) and higher family economic status (OR = 0.92, 95% CI [0.78–1.08]), were not significantly associated with suicidal ideation.

For suicide attempts, no protective factors were identified as statistically significant in the available studies.

### Publication bias and sensitivity analyses

Potential publication bias was examined using funnel plots and Egger’s regression tests. For predictors with sufficient numbers of studies, Egger’s regression tests did not indicate significant publication bias (*p* > 0.05), and visual inspection of funnel plots did not reveal substantial asymmetry. Funnel plots are presented in Supplementary materials 7 and 8.

Sensitivity analyses were conducted using a leave-one-out approach. The pooled effect sizes for several predictors showed some variation when individual studies were sequentially excluded, indicating that certain associations may be influenced by specific studies. However, the overall direction of the pooled estimates remained largely unchanged across sensitivity analyses.

### Moderator analyses

Substantial between-study heterogeneity was observed across predictors of suicidal ideation (I² range: 0%–98.76%) and suicide attempts (I² range: 0%–91.21%), indicating considerable variability in pooled effect sizes across studies. High levels of heterogeneity were observed for several predictors. For suicidal ideation, particularly high heterogeneity was observed for resilience (I² = 98.00%) and social support (I² = 98.76%), as well as for depressive symptoms (I² = 81.98%) and childhood emotional abuse (I² = 88.84%). Sleep-related predictors, including sleep duration, insomnia symptoms, and early morning awakening, also showed substantial heterogeneity across studies.

**Fig. 2:**
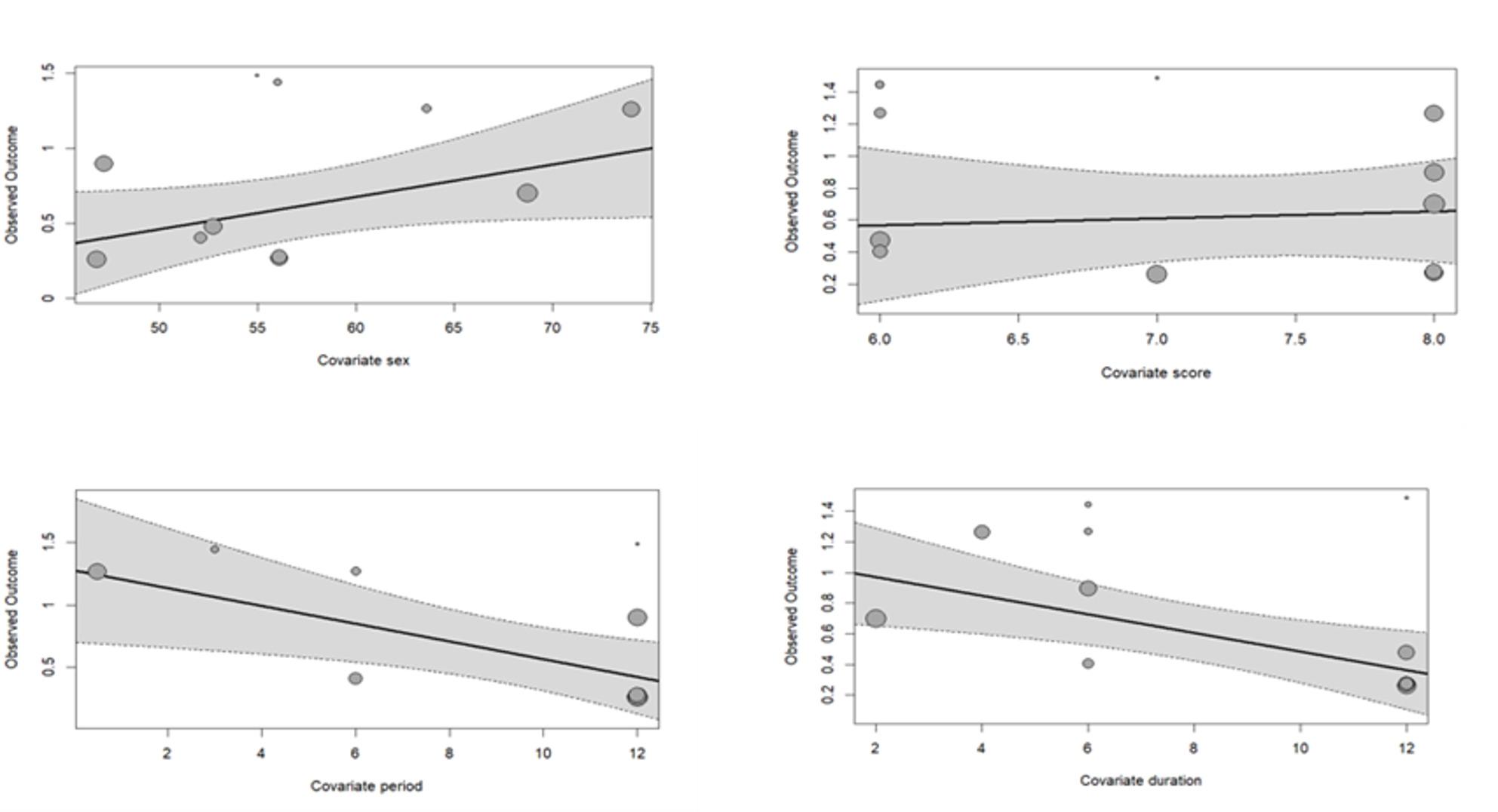
Moderator analyses via bubble plots: depressive symptoms predicting suicidal ideation

Meta-regression analyses were conducted for predictors with sufficient data (≥ 10 independent effect sizes), and the detailed results are presented in Table 3. For depressive symptoms and suicidal ideation, two temporal moderators showed significant effects (Table 3). Longer follow-up duration was associated with smaller pooled effect sizes (β = − 0.061, 95% CI [–0.105, − 0.007], *P* < 0.01), and later measurement periods were also associated with reduced effect sizes (β = − 0.071, 95% CI [–0.128, − 0.015], *P* < 0.05). The proportion of female participants was not significantly associated with effect size, and study quality was also not significantly associated with effect size. Bubble plots illustrating these associations are presented in Fig. 2.

**Table 3:** Moderators of the predictors of suicide ideation

Predictor	Moderator	K	β	95%CI	*P*	*R*²
Depressive symptoms	Sex (% female)	11	0.022	[–0.001, 0.045]	0.066	27.47%
	Follow-up duration (months)	11	–0.061	[–0.105, − 0.007]	< 0.01	54.72%
	Measure the suicidal ideation in which time period(month)	9	–0.071	[–0.128, − 0.015]	< 0.05	55.65%
	Quality score	11	0.043	[–0.251, 0.337]	0.774	0

*k*= Number of independent samples analyzed; 95%CI = 95% confidence interval of pooled OR; *β =* Standardized meta-regression coefficient, reflecting the direction and magnitude of the moderator’s effect on the association between the predictor (e.g., depressive symptoms) and suicidal ideation. Positive values indicate a stronger positive association, while negative values indicate a weaker or inverse association; *p* = p-value (two-tailed); R²=Proportion of between-study variance explained by the moderator in the meta-regression model (range: 0–100%), with higher values indicating greater explanatory power

## Discussion

Suicide remains a critical public health concern among university students worldwide, with relatively high prevalence rates of suicidal ideation and behaviors reported in Asian populations [16]. The university years constitute a particularly sensitive developmental period for suicide risk in the Chinese context, characterized by rapid social transitions, academic demands, and evolving family expectations. Improving understanding of risk and protective factors associated with suicidal ideation and suicide attempts among Chinese university students is therefore essential for timely identification and effective prevention.

To our knowledge, this study is among the first systematic reviews and meta-analyses to comprehensively synthesize longitudinal evidence on predictors of both suicidal ideation and suicide attempts among Chinese university students. By incorporating both English- and Chinese-language publications, this review extends prior work and captures culturally relevant evidence that has been underrepresented in the international literature. Moreover, the exclusive focus on longitudinal studies allows for a clearer examination of temporal relationships between predictors and suicidal outcomes than cross-sectional research.

Overall, our findings indicate that suicidal ideation and suicide attempts among Chinese university students are associated with factors across multiple domains, including behavioral and health-related factors, individual psychological characteristics, and interpersonal and sociocultural contexts. Several predictors emerged as particularly salient within this population, including sleep disturbances, prior suicidal ideation, psychological distress (e.g., depressive symptoms and anxiety), non-suicidal self-injury, and academic pressure. At the same time, examining how interpersonal and contextual factors—such as family functioning and academic stress—operate in this setting may provide useful insights for evaluating the cross-cultural applicability of dominant theories of suicidal behavior.

The following sections discuss these findings in greater detail across key domains of risk and protective factors.

### Risk factors for suicidal ideation and suicide attempts

#### Demographic factors

The meta-analytic results indicated that demographic characteristics showed limited predictive utility for suicidal ideation and suicide attempts among Chinese university students. Female sex was not significantly associated with suicidal outcomes in the pooled analyses, and residence location (urban vs. town) similarly did not demonstrate a significant association. These findings suggest that, within this population, demographic characteristics alone may have limited value in differentiating suicide risk.

This pattern differs somewhat from findings reported in some studies conducted in Western student populations, where certain demographic variables have shown stronger associations with suicidality [12, 15]. Several contextual factors may contribute to this difference. For example, the influence of academic stress—identified as a recurrent factor across several analyses in the present study—may reduce the relative contribution of demographic differences to suicide risk. In addition, rapid urbanization in China has narrowed traditional distinctions between rural and urban identities, particularly among university students who often move between multiple social and family environments [29].

Taken together, these findings suggest that suicide risk among Chinese university students may be more strongly associated with behavioral and psychological indicators than with demographic characteristics alone. From a prevention perspective, this pattern highlights the potential importance of focusing on modifiable risk factors, such as sleep problems, psychological distress, and academic stress.

#### Behavioral and health-related factors

This meta-analysis provides longitudinal evidence that sleep disturbances are important predictors of suicidal ideation and suicide attempts among Chinese university students. Early morning awakening was associated with suicidal ideation, while insomnia symptoms were associated with both suicidal ideation and suicide attempts. These findings are consistent with a growing body of international research identifying sleep disturbances as transdiagnostic risk factors across the suicide continuum [12, 30]. Recent work using multidimensional risk profiling among university students has also highlighted sleep problems as a central component of suicide risk patterns [31].

Several mechanisms may help explain these associations. Sleep disturbances may impair emotional regulation and stress tolerance, potentially increasing vulnerability to suicidal thoughts and behaviors [32, 33]. Beyond biological pathways, contextual and institutional factors may also play a role. In some Chinese universities, early class schedules may require students to engage in cognitively demanding activities during circadian low points, which could potentially exacerbate the effects of sleep deprivation [34]. Collectively, these findings suggest that sleep health may represent a potentially modifiable and relatively low-stigma target for suicide prevention. Interventions such as cognitive behavioral therapy for insomnia and campus-level strategies aimed at improving sleep health may warrant further investigation in future intervention studies [35].

Alcohol use also emerged as a significant behavioral risk factor, although the observed association appeared somewhat smaller than those reported in some Western student populations [36]. This difference may partly reflect variations in drinking patterns across student populations, as Chinese university students generally report lower rates of binge drinking [37]. Nevertheless, alcohol-related disinhibition during periods of heightened stress may still increase vulnerability to suicidal behaviors, highlighting the potential value of prevention strategies that address both sleep health and substance use.

#### Individual psychological factors

Among individual psychological factors, several variables were significantly associated with suicidal ideation in the pooled analyses. Prior suicidal ideation showed the strongest association with subsequent suicidal ideation, suggesting substantial temporal persistence of suicidal thoughts within this population. Anxiety and depressive symptoms were also significantly associated with suicidal ideation. These findings are broadly consistent with international research indicating that psychological distress represents a central vulnerability for the development of suicidal thoughts among university students [38].

In contrast, some psychological variables did not demonstrate significant pooled associations with suicidal ideation in the present analysis, including prior suicide attempts and suboptimal health status. Previous studies have reported mixed findings regarding the predictive value of these variables, and differences in measurement approaches or follow-up durations across studies may partly contribute to this variability.

For suicide attempts, a more restricted set of psychological predictors emerged. Prior non-suicidal self-injury showed a significant association with suicide attempts, whereas several factors that were significantly associated with suicidal ideation—including depressive symptoms, prior suicidal ideation, and history of mental illness—did not reach statistical significance in the pooled analyses of suicide attempts.

This pattern is partly consistent with international research, which also suggests that psychological distress is more strongly associated with suicidal ideation than with suicide attempts [39]. However, some discrepancies were observed. For example, depressive symptoms and history of mental illness have been reported as significant predictors of suicide attempts in certain international studies, whereas they were not statistically significant in the present analysis.

Several factors may contribute to these differences. First, the present review focused exclusively on non-clinical university student populations and excluded psychiatric or hospital-based samples. As a result, the overall severity of mental disorders may have been lower than in clinical populations, potentially attenuating the observed associations for some psychiatric risk factors. Second, variability in measurement approaches and statistical adjustment strategies across studies may have influenced the comparability of effect estimates. Third, sociocultural contextual factors may also play a role, although these mechanisms were not directly examined in the included studies. In addition, the number of available longitudinal studies examining predictors of suicide attempts among university students remains relatively limited, which may reduce statistical power.

From a theoretical perspective, these findings are consistent with ideation-to-action frameworks of suicidal behavior, which propose that factors contributing to the emergence of suicidal ideation may differ from those involved in the transition from ideation to suicidal behavior [40]. In this perspective, psychological distress factors such as depressive symptoms and anxiety may play a central role in the development of suicidal thoughts, whereas the progression to suicide attempts may depend more strongly on factors that increase capability for suicide, such as exposure to painful or fear-inducing experiences [41, 42]. The observed association between non-suicidal self-injury and suicide attempts in the present analysis is consistent with this theoretical perspective and aligns with the acquired capability component of the interpersonal–psychological theory of suicide [24].

#### Interpersonal and sociocultural factors

Academic pressure emerged as a significant predictor of both suicidal ideation and suicide attempts in the pooled analyses. Previous international studies have also identified academic stress as an important correlate of suicidal behaviors among students, although the magnitude of this association varies across educational systems and cultural contexts. In China, educational achievement often carries substantial personal and familial expectations, and perceived academic failure may be associated with feelings of shame, hopelessness, or social pressure [43]. These contextual factors may be relevant to understanding the observed association between academic pressure and suicidal outcomes, although the underlying mechanisms were not directly examined in the included studies. Therefore, these interpretations should be considered as contextual hypotheses and interpreted with caution.

Adverse life events were also significantly associated with suicidal ideation, consistent with international evidence linking stressful experiences to increased suicide risk [44]. Stressful life events may disrupt coping resources and increase psychological vulnerability, particularly during periods of developmental transition such as university entry. As potentially modifiable stressors, adverse life events highlight the importance of prevention strategies that extend beyond risk identification to include timely psychosocial support.

Poor family functioning also emerged as a significant interpersonal risk factor for suicidal ideation. Family environments characterized by conflict, low emotional support, or communication difficulties may contribute to feelings of isolation or distress among students during critical developmental periods [45]. These findings align with broader literature emphasizing the role of family relationships in shaping mental health outcomes among young adults.

### Protective factors for suicidal ideation and suicide attempts

Social support was the protective factor that reached statistical significance among the limited set of protective variables examined in this meta-analysis, which also included resilience and family economic status. This finding should be interpreted cautiously, as it may partly reflect the limited number of protective factors examined in the available longitudinal literature rather than indicating that social support represents the sole protective resource.

In addition, measurement considerations may have influenced these results. Many instruments used to assess psychosocial resources were adapted from Western contexts and may not fully capture culturally specific forms of coping or social support among Chinese university students. Consequently, other potentially important protective factors may remain underrepresented in the current evidence base.

Some contextual factors may also influence the observable protective effects of social support. For example, concerns about stigma, reluctance to disclose emotional distress, or norms surrounding self-reliance may affect help-seeking behaviors among students [46]. However, because these mechanisms were not directly examined in the included studies, such interpretations should be considered cautiously.

### Meta-regression and temporal dynamics

Meta-regression analyses suggested a potential temporal pattern in the association between depressive symptoms and suicidal ideation. Specifically, the magnitude of the association appeared to decrease as follow-up duration increased, indicating that depressive symptoms may be more strongly related to suicidal ideation in shorter follow-up intervals. Similar temporal dynamics have been discussed in the literature, where depressive symptoms may represent a background vulnerability while more proximal stressors contribute to the emergence of suicidal crises [47, 48]. However, this interpretation should be considered cautiously because meta-regression analyses are observational and may be influenced by differences in study design, measurement, and follow-up intervals across the included studies.

### Limitations and strengths

Several limitations should be considered when interpreting the findings of this meta-analysis. First, variation in the measurement of suicidal ideation and suicide attempts across studies may have introduced additional heterogeneity into the pooled estimates. The included studies employed different instruments and operational definitions, ranging from brief self-report items to standardized psychological scales, which may capture different dimensions or levels of severity of suicidal thoughts and behaviors.

Second, substantial statistical heterogeneity was observed in several analyses. Although variation in outcome measurement may partly explain this heterogeneity, broader differences in measurement and methodology across studies are also likely to have contributed. For example, constructs were assessed using different instruments or Chinese-adapted versions of internationally developed scales, which may vary in translation, cultural adaptation, and scale versions. In addition, predictors were operationalized inconsistently, with some treated as categorical variables using predefined cut-off scores and others analyzed as continuous measures. Methodological differences may have further contributed to heterogeneity. Although adjusted odds ratios were prioritized, the covariates included in multivariable models differed across studies. Differences in sample characteristics and follow-up durations may also have influenced the observed variability. Such heterogeneity is common in meta-analyses of psychosocial risk factors and may limit the precision and generalizability of pooled effect estimates. Therefore, findings for predictors with particularly high heterogeneity should be interpreted with caution.

Third, most included studies relied on self-report instruments, which may be subject to response bias. Under-reporting of suicidal thoughts or behaviors may occur due to social desirability or concerns related to mental health stigma, which could influence the accuracy of reported associations. In addition, although all included studies employed longitudinal designs, the evidence remains observational in nature. While longitudinal studies strengthen temporal inference, they do not establish causal relationships. Therefore, the findings should be interpreted as longitudinal associations rather than causal effects, and residual confounding and unmeasured factors may still influence the observed relationships.

Fourth, the available longitudinal evidence on predictors of suicide attempts and protective factors among university students remains relatively limited. As a result, some pooled estimates were based on a relatively small number of studies, and more detailed subgroup analyses to explore sources of heterogeneity were not always feasible. Future large-scale longitudinal studies examining a broader range of risk and protective factors would help strengthen the evidence base in this area.

Despite these limitations, this study has several notable strengths. To our knowledge, this is among the first systematic review and meta-analysis to synthesize longitudinal evidence on both risk and protective factors for suicidal ideation and suicide attempts specifically among Chinese university students. By focusing exclusively on longitudinal studies and incorporating both English- and Chinese-language publications, the present analysis strengthens temporal inference and provides a more comprehensive synthesis of the available evidence. In addition, the distinction between predictors of suicidal ideation and suicide attempts allows for a more nuanced interpretation of suicide risk processes within this population.

### Implications for practice and research

The findings of this meta-analysis may have implications for suicide prevention efforts in higher education settings. The transition to university represents a potentially vulnerable developmental period, and improving the early identification of modifiable risk factors may help support timely prevention efforts. By synthesizing longitudinal evidence, this study provides a foundation for understanding suicide risk trajectories and identifying factors that may inform prevention strategies at both individual and institutional levels.

Our results highlight the importance of considering both universal and contextually relevant risk factors within multilevel prevention frameworks. In recent years, China has introduced several policy initiatives aimed at strengthening mental health services for university students. For example, the Healthy China 2030 Plan emphasizes the expansion of mental health services, and the Guidelines for Mental Health Education for University Students encourage universities to establish counseling centers, integrate mental health education into curricula, and implement early identification systems for psychological difficulties. By 2025, most universities in China had established counseling services, and routine psychological screening for incoming students has become increasingly common, providing an important context for interpreting the present findings for an international readership.

Despite these policy developments, challenges remain in translating policy frameworks into consistent practice across institutions. The availability of counseling resources varies substantially across universities, and referral pathways and follow-up support mechanisms following psychological screening may not always be consistently implemented. In addition, many intervention approaches have been adapted from Western contexts, and their applicability within Chinese university settings may require further evaluation.

In this context, the findings of this review suggest several potential directions for strengthening suicide prevention in higher education environments. At the institutional level, universities may consider strategies aimed at addressing modifiable risk factors identified in this review, such as sleep disturbances and academic stress. Approaches that promote mental health literacy, strengthen campus support systems, and improve access to psychological services may help address the identified gaps between policy and practice. However, these strategies should be considered as areas for further evaluation rather than established solutions.

Future research should prioritize the development of culturally validated assessment tools that better reflect the language and lived experiences of Chinese university students. Large-scale longitudinal studies across different types of universities and academic disciplines would further improve understanding of suicide risk trajectories in this population. In addition, rigorous evaluations of culturally responsive prevention and intervention strategies are needed. Integrating universal theories of suicidality with context-specific mechanisms may help refine both theoretical models and practical applications. Ultimately, reducing suicide risk among Chinese university students will likely require coordinated efforts across individual, institutional, and societal levels to support both psychological well-being and academic development.

## Conclusion

This meta-analysis synthesizes longitudinal evidence on risk and protective factors for suicidal ideation and suicide attempts among Chinese university students. Several psychological factors, including prior suicidal ideation, depressive symptoms, anxiety, and history of mental illness, were significantly associated with suicidal ideation, consistent with findings reported in international research. In addition, sleep disturbances, academic pressure, and poor family functioning emerged as important contextual correlates of suicidal outcomes in this population.

The findings also suggest that social support may play a protective role, although the evidence for protective factors remains limited. Other potential protective resources, such as resilience and family socioeconomic status, showed non-significant associations in the current analyses and warrant further investigation in future longitudinal studies.

Overall, these results highlight the importance of considering both psychological vulnerabilities and contextual stressors when examining suicide risk among university students. Future research should continue to investigate culturally relevant mechanisms of suicide risk and evaluate prevention strategies that address modifiable risk factors within university settings.

## Supplementary Information

Supplementary Material 1.

Supplementary Material 2.

Supplementary Material 3.

Supplementary Material 4.

Supplementary Material 5.

Supplementary Material 6.

Supplementary Material 7.

Supplementary Material 8.

Supplementary Material 9.

## Data Availability

All data analyzed in this study were obtained from previously published studies. The datasets used and/or analyzed during the current study are available from the corresponding author on reasonable request.
