# Supplementary material for: Risk and protective factors for suicidal ideation and suicide attempts among Chinese university students: a systematic review and meta-analysis of longitudinal studies
Source: BMC Public Health. 2026 Apr 20;26:1787. doi: 10.1186/s12889-026-27430-0 (PMC13235143; doi:10.1186/s12889-026-27430-0)
Supplement: Supplementary file 2 — Supplementary Material 2. [file 12889_2026_27430_MOESM2_ESM.docx]

# **Supplementary material 2** Search strategy

| Database | Query |
| --- | --- |
| Web of Science | ("China" OR "Chinese") AND ("college student" OR "undergraduate*" OR "freshman*") AND (suicide OR suicidality OR suicidal behavior OR suicid* thought* OR suicid* ideation* OR suicid* attempt* OR suicide death OR suicide plan OR suicide gesture OR self-injury OR self-harm OR self-destruction OR self-mutilation OR self-inflicted OR self-burning OR self-cutting OR nonsuicidal self-injury OR nonsuicidal self-injury) |
| PubMed | ("China" OR "Chinese") AND ("college student" OR "undergraduate*" OR "freshman*") AND (suicide OR suicidality OR suicidal behavior OR suicid* thought* OR suicid* ideation* OR suicid* attempt* OR suicide death OR suicide plan OR suicide gesture OR self-injury OR self-harm OR self-destruction OR self-mutilation OR self-inflicted OR self-burning OR self-cutting OR nonsuicidal self-injury OR nonsuicidal self-injury) |
| PsycINFO | ("China" OR "Chinese") AND ("college student" OR "undergraduate*" OR "freshman*") AND (suicide OR suicidality OR suicidal behavior OR suicid* thought* OR suicid* ideation* OR suicid* attempt* OR suicide death OR suicide plan OR suicide gesture OR self-injury OR self-harm OR self-destruction OR self-mutilation OR self-inflicted OR self-burning OR self-cutting OR nonsuicidal self-injury OR nonsuicidal self-injury) |
| CNKI（Chinese） | （大学 + 大学生 + 大学新生 + 高职生 + 高校学生）and 自杀 and （纵向 + 预测 + 追踪 + 队列 + 随访 + 病例） |
| Wanfang（Chinese） | (摘要:(（大学 or 大学生 or 高校学生 or 高职生 or 大学新生）) and 摘要:("自杀") and 摘要:(（追踪 or 预测 or 纵向 or 队列 or 随访 or 病例）)) |
| Weipu（Chinese） | （大学生 or 大学 or 高校学生 or 高职生 or 大学新生）and 自杀 and （追踪 or 预测 or 纵向 or 队列 or 随访 or 病例） |
