# Supplementary material for: Risk and protective factors for suicidal ideation and suicide attempts among Chinese university students: a systematic review and meta-analysis of longitudinal studies
Source: BMC Public Health. 2026 Apr 20;26:1787. doi: 10.1186/s12889-026-27430-0 (PMC13235143; doi:10.1186/s12889-026-27430-0)
Supplement: Supplementary file 3 — Supplementary Material 3. [file 12889_2026_27430_MOESM3_ESM.docx]

# **Supplementary material 3** Characteristics of 22 longitudinal studies included for systematic review

| No. | Author (year) | Follow-up duration (months) | Sample  size | Age  (years) | Sex  (%female) | Outcome | Inventory of outcome | Risk factors | NOS score | Meta-analysis  included |
| --- | --- | --- | --- | --- | --- | --- | --- | --- | --- | --- |
| 1.1 | Cao et al., 2012 | 6 | 3460 | 18.77  (mean) | 51.2 | suicidal ideation | self-report | female, region, residence location, parental marital status, family economic status, crushing academic workload, personalities, cigarette smoking, alcohol use, prior suicide ideation, prior suicide attempts, prior NSSI, somatic pathological symptoms, psychopathological symptoms | 6 | Yes |
| 1.2 | Cao et al., 2012 | 6 | 3460 | 18.77  (mean) | 51.2 | suicide attempts | self-report | female, region, residence location, parental marital status, family economic status, academic pressure, personalities, cigarette smoking, alcohol use, prior suicide ideation, prior suicide attempts, prior NSSI, somatic pathological symptoms, psychopathological symptoms | 6 | Yes |
| 2.1 | Teng et al., 2018 | 24 | 2291 | ≥18 | 64.94 | suicidal ideation | self-report | history of mental illness, attitudinal acceptance of suicide, prior suicide ideation, prior suicide attempt | 7 | Yes |
| 2.2 | Teng et al., 2018 | 24 | 2291 | ≥18 | 64.94 | suicide attempts | self-report | physical illness, history of mental illness, acceptance of suicide, prior suicide ideation, prior suicide attempts | 7 | Yes |
| 3.1 | Xing et al., 2010 | 3 | 1658 | ≥18 | 52.17 | suicidal ideation | SQPMHA-CV | academic pressure, suboptimal health status | 6 | Yes |
| 3.2 | Xing et al., 2010 | 3 | 1658 | ≥18 | 52.17 | suicide attempts | SQPMHA-CV | academic pressure, suboptimal health status | 6 | Yes |
| 4 | Fu et al., 2022 | 6 | 558 | ≥18 | 56 | suicidal ideation | BSS | depression, internet bullying | 6 | Yes |
| 5 | Tu et al., 2005 | 6 | 2075 | ≥18 | 52.1 | suicidal ideation | self-report | suboptimal health status, residence location, personalities, unhealthy weight loss, alcohol use, only-children family, body type (medium, sideways, thin), anxiety symptoms, depression | 6 | Yes |
| 6 | Wang et al., 2024 | 12 | 1145 | 18.8 | 75.2 | suicidal ideation | SIOSS | female, only-children family, residence location, family economic status, parental marital status, parental relationship (good, poor, fair), childhood life (happy, average, unfortunate), prior suicide attempts, extraversion, neuroticism, psychoticism | 7 | Yes |
| 7.1 | Yanglinsheng, 2013 | 12 | 7298 | 19.33 | 56.1 | suicidal ideation | self-report | prior suicide ideation, negative life events, number of adverse experiences, religion, female, self-aggression, depression, family function | 8 | Yes |
| 7.2 | Yanglinsheng, 2013 | 12 | 7298 | 19.33 | 56.1 | suicide attempts | self-report | prior suicide ideation, prior suicide attempts, negative life events, self-aggression, female | 8 | Yes |
| 8 | Zhang& Chen, 2021 | 48 | 5372 | ≥18 | 30.6 | suicidal ideation | PANSI | female、residence location | 6 | Yes |
| 9 | Huang et al., 2022 | 4 | 35516 | ≥18 | 74 | suicidal ideation | PHQ-9 | female, sophomore, junior, senior, postgraduate, urban, only-children family, history of mental illness, confirmed COVID-19 cases in the community or village, relatives or friends being infected with COVID-19, depression, social support, positive coping, negative coping, family function | 8 | Yes |
| 10.1 | Li et al., 2024 | 19 | 4326 | 18.63 | 48.2 | suicidal ideation | self-report | insomnia symptoms, resilience | 7 | Yes |
| 10.2 | Li et al., 2024 | 19 | 4326 | 18.63 | 48.2 | suicide attempts | self-report | insomnia symptoms, resilience | 7 | Yes |
| 11 | Liu et al., 2023 | 6 | 1897 | 19.93 | 57.1 | suicidal ideation | HDSQ | perceived discrimination, female, age, GPA, impoverished students | 6 | Yes |
| 12 | Liu et al., 2024 | 6 | 674 | 19.96 | 63.6 | suicidal ideation | HDSQ | negative perfectionism, positive perfectionism, sex, male, Age, Negative life events, depression | 6 | Yes |
| 13 | Ma et al., 2022 | 2 | 67905 | 20.19 | 68.7 | suicidal ideation | PHQ-9 | depression, anxiety, acute stress symptoms, obsessive-compulsive symptoms, insomnia | 8 | Yes |
| 14.1 | Qiao et al., 2023 | 6 | 3834 | 19.12 | 47.2 | suicidal ideation | SBQ-R | insomnia symptoms, depressive symptoms, NSSI | 8 | Yes |
| 14.2 | Qiao et al., 2023 | 6 | 3834 | 19.12 | 47.2 | suicide attempts | SBQ-R | insomnia symptoms, depressive symptoms, NSSI | 8 | Yes |
| 15.1 | Shi et al., 2022 | 12 | 3871 | 19.13 | 46.8 | suicidal ideation | SBQ-R | NSSI, depressive symptoms, hopelessness, trait impulsivity, childhood emotional abuse, family conflict, reasons for living | 7 | Yes |
| 15.2 | Shi et al., 2022 | 12 | 3871 | 19.13 | 46.8 | suicide attempts | SBQ-R | NSSI, depressive symptoms, hopelessness, trait impulsivity, childhood emotional abuse, family conflict, reasons for living | 7 | Yes |
| 16.1 | Shi et al., 2020 | 6 | 10338 | 20.15 | 49.9 | suicidal ideation | self-report | insomnia, nightmares, SDB symptoms, RLS symptoms | 8 | Yes |
| 16.2 | Shi et al., 2020 | 6 | 10338 | 20.15 | 49.9 | suicide attempts | self-report | insomnia, nightmares, SDB symptoms, RLS symptoms | 8 | Yes |
| 17 | Sun et al., 2023 | 6 | 910 | ≥18 | NR | suicidal ideation | PQPHE | age, female, ethnicity, residence location, family income, parental marital status, “Left-behind” child status, single child status, history of mental disorders, chronic physical illness, psychotic-like experiences (PLEs, childhood trauma, resilience, social support | 6 | Yes |
| 18 | Wang et al., 2021 | 1 | 67905 | 20.23 | 68.7 | suicidal ideation | PHQ-9 | sleep duration, difficulty initiating sleep, difficulty maintaining sleep, early morning awakening, subjective sleep quality, sleep disturbance | 8 | Yes |
| 19 | Xu et al., 2022 | 6 | 197 | ≥18 | 58.9 | suicidal ideation | SIOSS | perceived stress, sleep quality | 6 | Yes |
| 20 | Yang et al., 2020 | 12 | 1361 | 20.46 | 52.76 | suicidal ideation | SIOSS | stress, trait anticipatory anhedonia, trait consummatory anhedonia, state anhedonia, depression | 6 | Yes |
| 21 | Zheng et al., 2024 | 9 | 333 | 19.41 | 61.26 | suicidal ideation | BDI-9 | total sleep time, morningness chronotype, eveningness chronotype | 7 | Yes |
| 22 | Wang et al., 2023 | 12 | 211 | 19.75 | 54.98 | suicidal ideation | self-report | female, major (clinical medicine), parents’ income, depression, loneliness, defeat, social support, interpersonal needs, entrapment | 7 | Yes |

*Note*. NOS score: Newcastle- Ottawa Scale score; NR: not reported. SQPMHA-CV: Self-Questionnaire on Physical-Mental Health for Adolescents - China Version；BSS: Beck Scale for Suicide Ideation; SIOSS: Self-Rating Inventory of Suicide Ideation Scale (SIOSS); PHQ-9: Patient Health Questionnaire-9 (PHQ-9); PANSI: Positive and Negative Suicidal Ideation; HDSQ: The 4- item suicidal ideation sub-questionnaire of the Hopeless Depression Questionnaire; SBQ-R: suicide behaviors questionnaire-revised; PQPHE: Psychological Questionnaire for Public Health Emergency; BDI: Beck Depression Inventory.
