# Supplementary material for: Risk and protective factors for suicidal ideation and suicide attempts among Chinese university students: a systematic review and meta-analysis of longitudinal studies
Source: BMC Public Health. 2026 Apr 20;26:1787. doi: 10.1186/s12889-026-27430-0 (PMC13235143; doi:10.1186/s12889-026-27430-0)
Supplement: Supplementary file 4 — Supplementary Material 4. [file 12889_2026_27430_MOESM4_ESM.docx]

# **Supplementary material 4** Quality assessment of included studies

|  | **Selection** | | | | **Comparability** | **Outcome** | | | **Overall** |
| --- | --- | --- | --- | --- | --- | --- | --- | --- | --- |
|  | 1 | 2 | 3 | 4 | 1 | 1 | 2 | 3 |  |
| Cao et al., 2012 | 0 | 1 | 1 | 1 | 1 | 1 | 1 | 0 | 6 |
| Teng et al., 2018 | 1 | 1 | 1 | 0 | 2 | 0 | 1 | 1 | 7 |
| Xing et al., 2010 | 1 | 1 | 1 | 0 | 1 | 1 | 1 | 0 | 6 |
| Fu et al., 2022 | 0 | 1 | 1 | 0 | 2 | 1 | 1 | 0 | 6 |
| Tu et al., 2005 | 1 | 0 | 1 | 0 | 2 | 0 | 1 | 1 | 6 |
| Wang et al., 2024 | 1 | 1 | 1 | 0 | 2 | 1 | 1 | 0 | 7 |
| Yanglinsheng, 2013 | 1 | 1 | 1 | 1 | 2 | 0 | 1 | 1 | 8 |
| Zhang& Chen, 2021 | 0 | 1 | 1 | 1 | 2 | 1 | 1 | 0 | 6 |
| Huang et al., 2022 | 1 | 1 | 1 | 1 | 2 | 0 | 1 | 1 | 8 |
| Li et al., 2024 | 0 | 1 | 1 | 1 | 2 | 1 | 1 | 0 | 7 |
| Liu et al., 2023 | 1 | 1 | 1 | 0 | 1 | 0 | 1 | 1 | 6 |
| Liu et al., 2024 | 1 | 1 | 0 | 0 | 2 | 1 | 0 | 1 | 6 |
| Ma et al., 2022 | 1 | 1 | 1 | 1 | 2 | 1 | 1 | 0 | 8 |
| Qiao et al., 2023 | 1 | 1 | 1 | 0 | 2 | 1 | 1 | 1 | 8 |
| Shi et al., 2022 | 0 | 1 | 1 | 1 | 2 | 1 | 1 | 0 | 7 |
| Shi et al., 2020 | 1 | 1 | 1 | 1 | 2 | 0 | 1 | 1 | 8 |
| Sun et al., 2023 | 1 | 1 | 0 | 0 | 2 | 1 | 1 | 0 | 6 |
| Wang et al., 2021 | 1 | 1 | 1 | 0 | 2 | 1 | 1 | 1 | 8 |
| Xu et al., 2022 | 1 | 1 | 0 | 0 | 2 | 1 | 1 | 0 | 6 |
| Yang et al., 2020 | 1 | 1 | 1 | 0 | 1 | 1 | 1 | 0 | 6 |
| Zheng et al., 2024 | 0 | 1 | 1 | 1 | 2 | 0 | 1 | 1 | 7 |
| Wang et al., 2023 | 1 | 1 | 1 | 0 | 1 | 1 | 1 | 1 | 7 |
