# Supplementary figures and images for: Risk and protective factors for suicidal ideation and suicide attempts among Chinese university students: a systematic review and meta-analysis of longitudinal studies
Source: BMC Public Health. 2026 Apr 20;26:1787. doi: 10.1186/s12889-026-27430-0 (PMC13235143; doi:10.1186/s12889-026-27430-0)

#
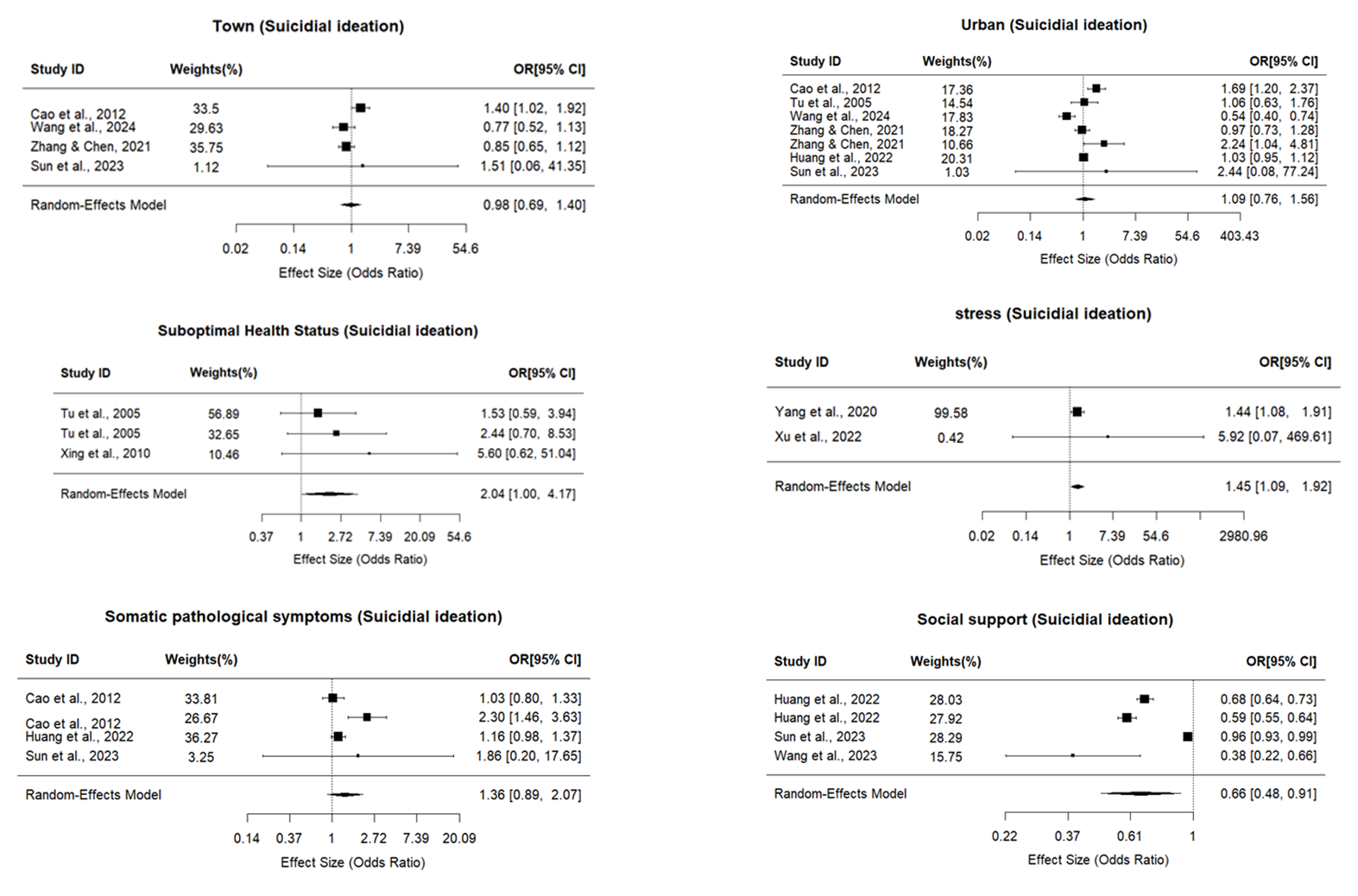
**Supplementary material 5** Forest plot of the predictors of suicidal ideation


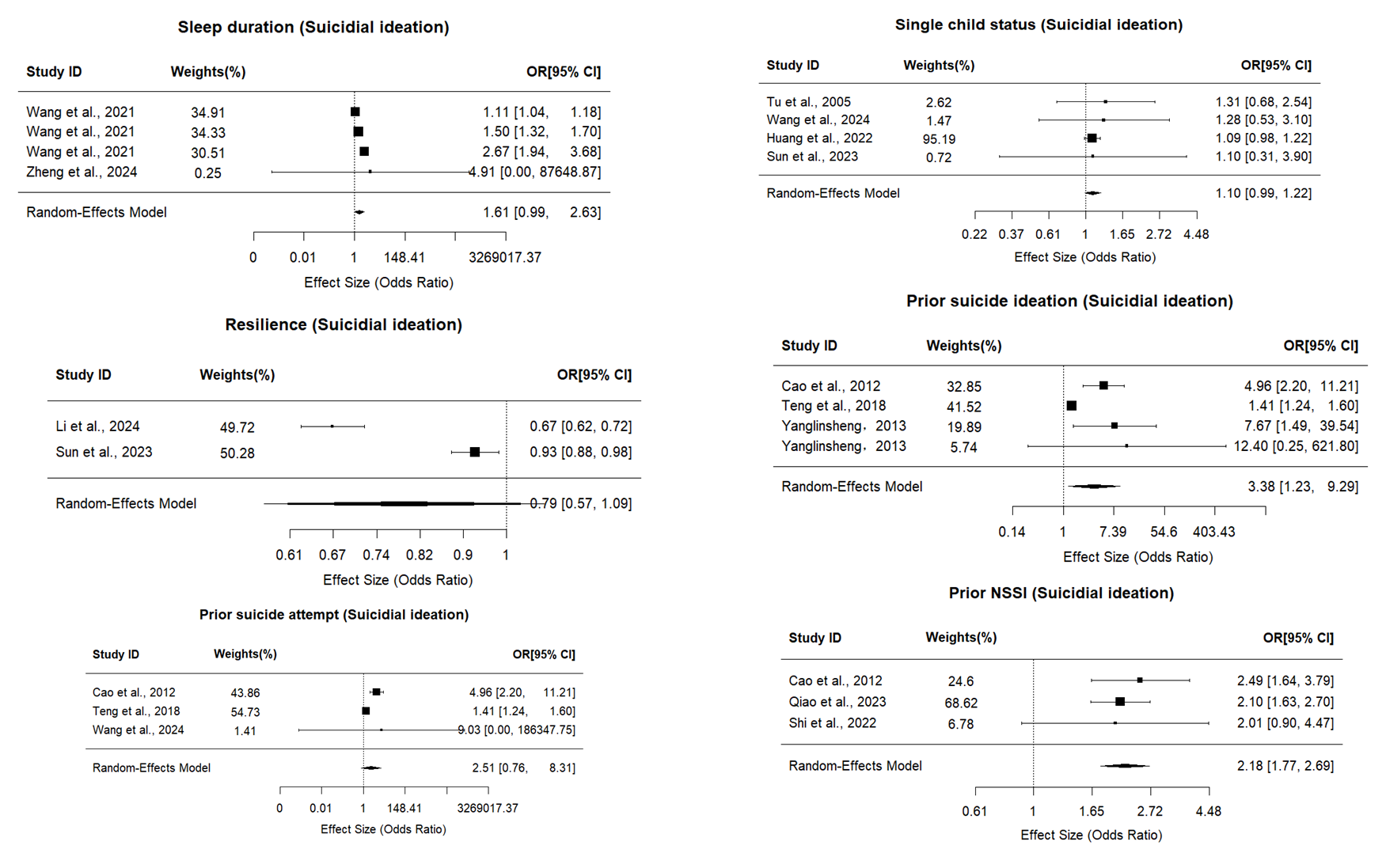


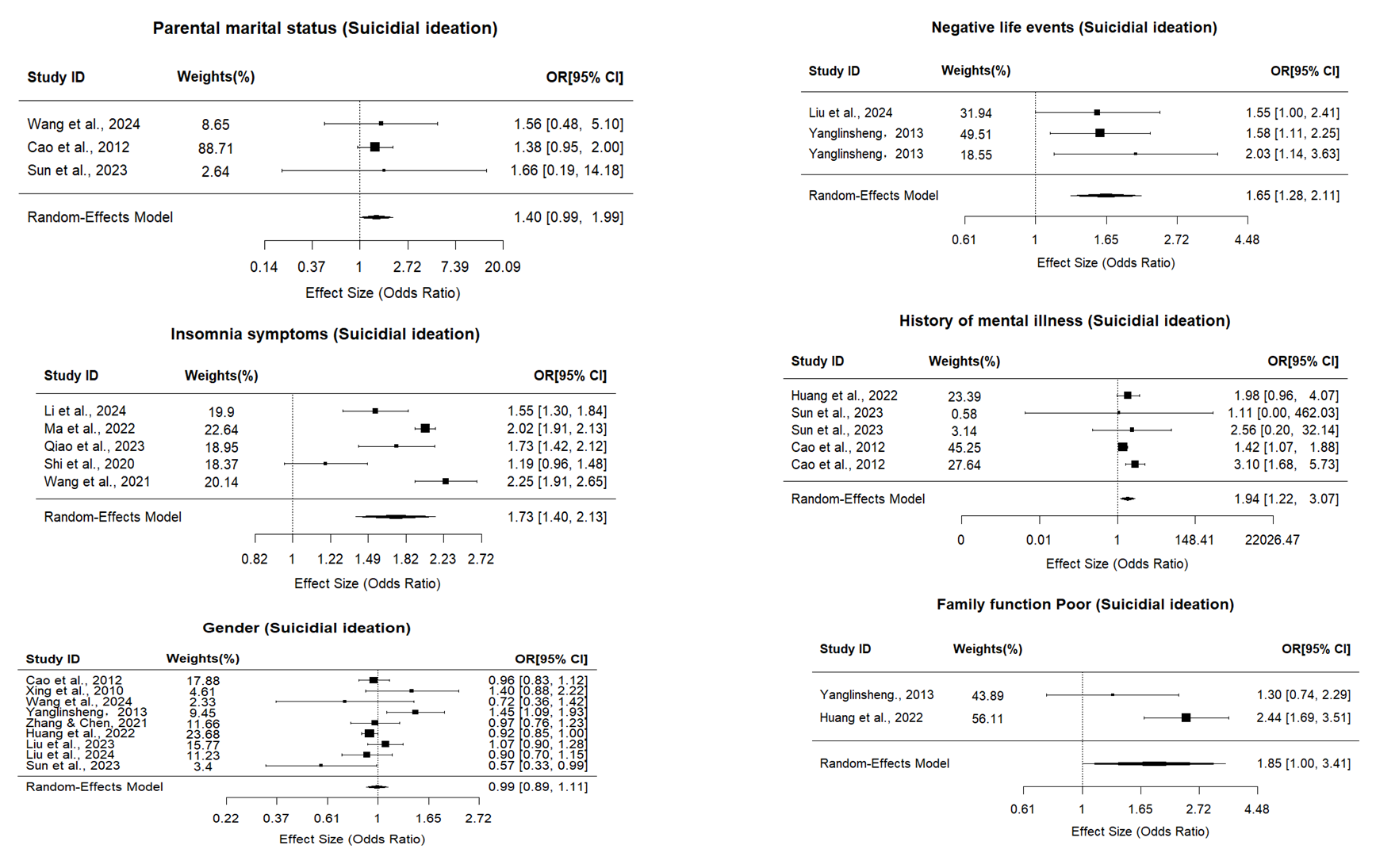


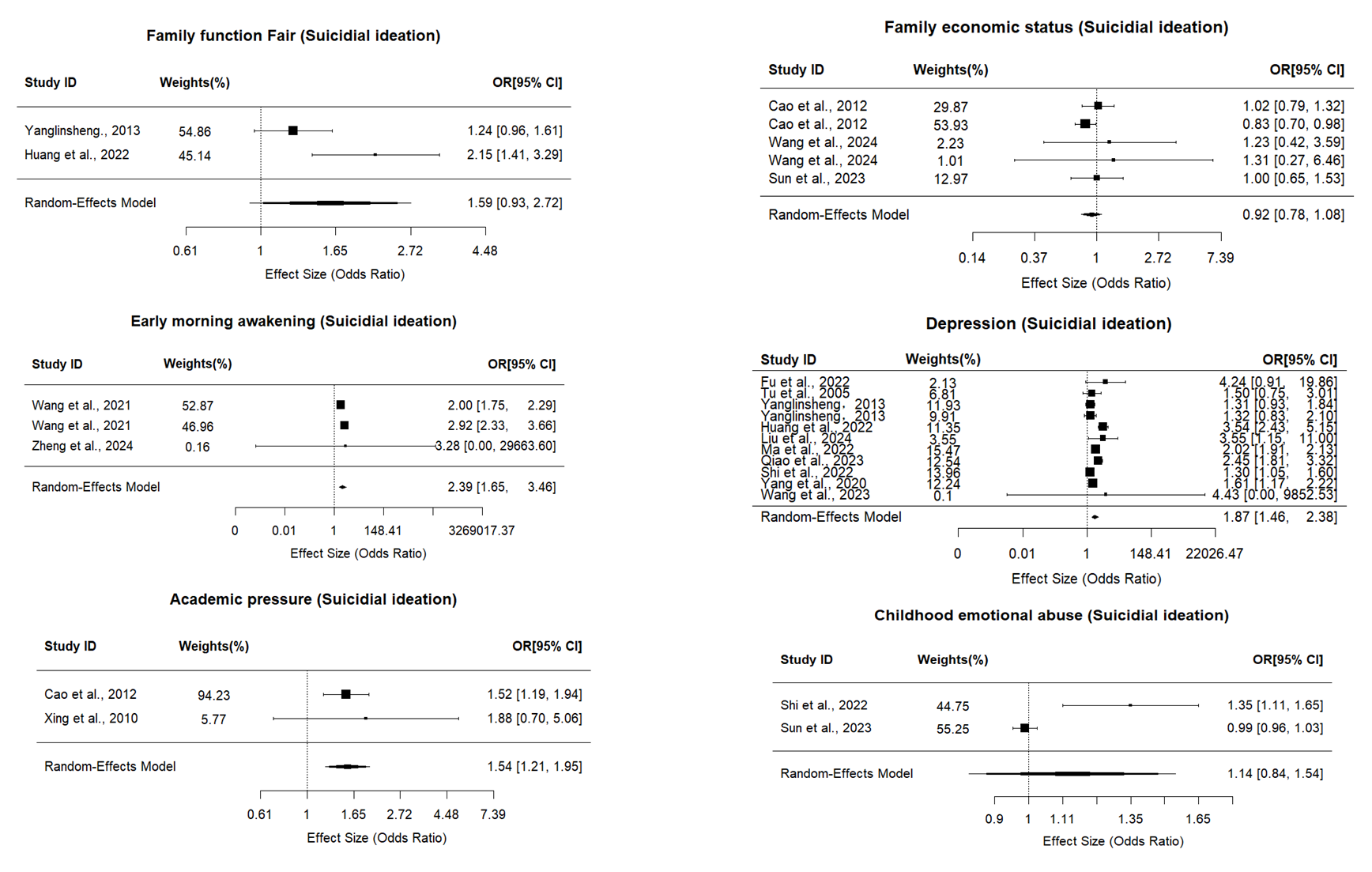


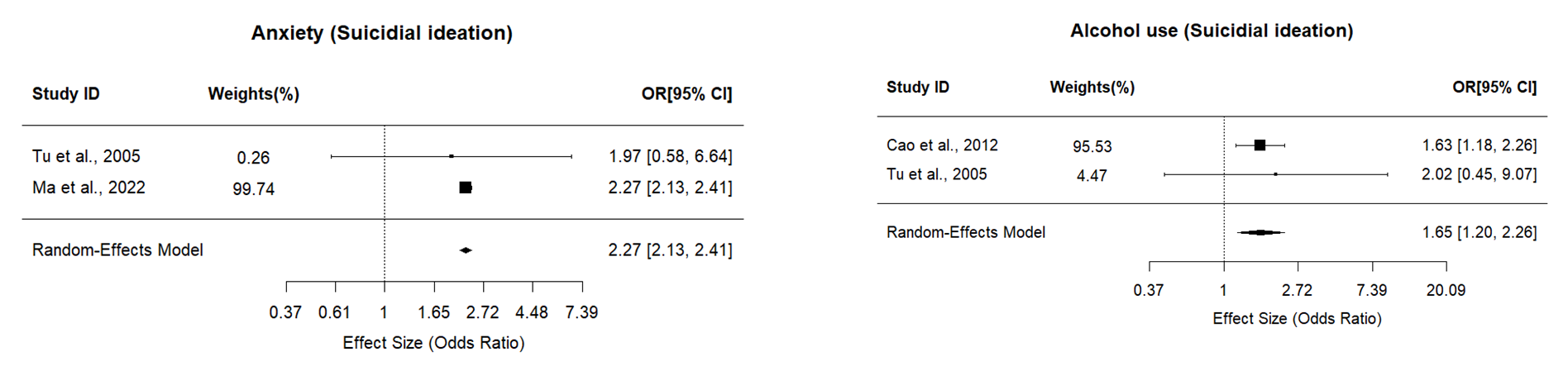

Supplement: Supplementary file 5 — Supplementary Material 5. [file 12889_2026_27430_MOESM5_ESM.docx]

# **Supplementary material 6** Forest plot of the predictors of suicide attempts


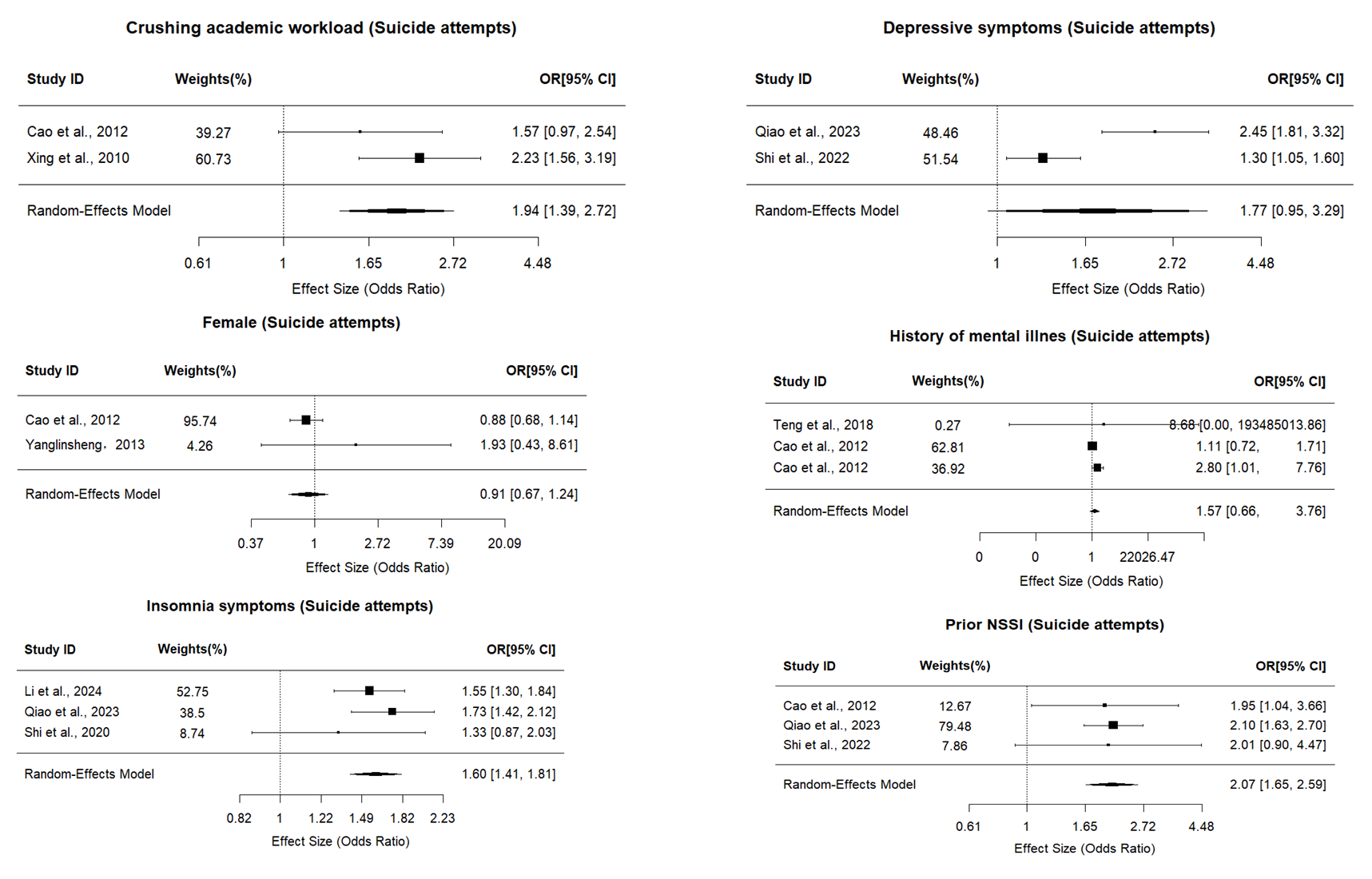


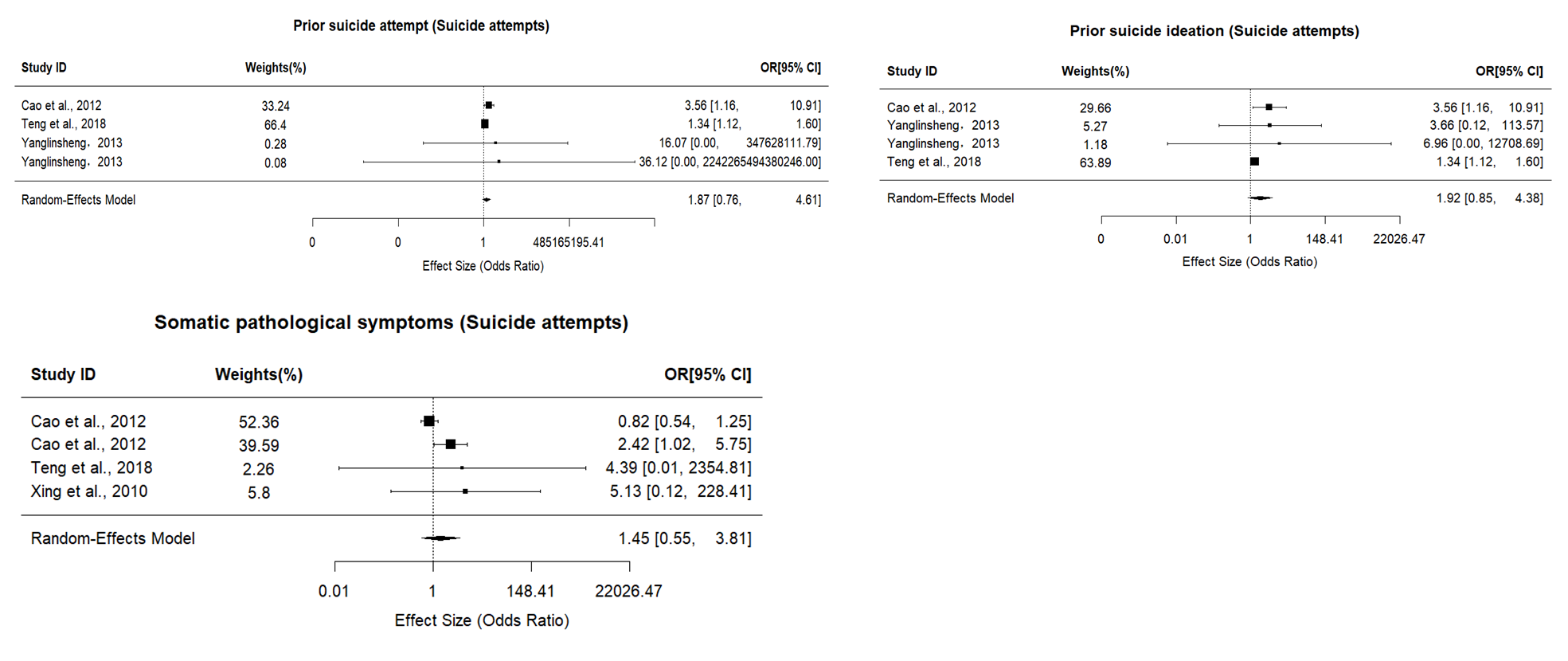

Supplement: Supplementary file 6 — Supplementary Material 6. [file 12889_2026_27430_MOESM6_ESM.docx]

# **Supplementary material 7** Funnel plot of suicidal ideation


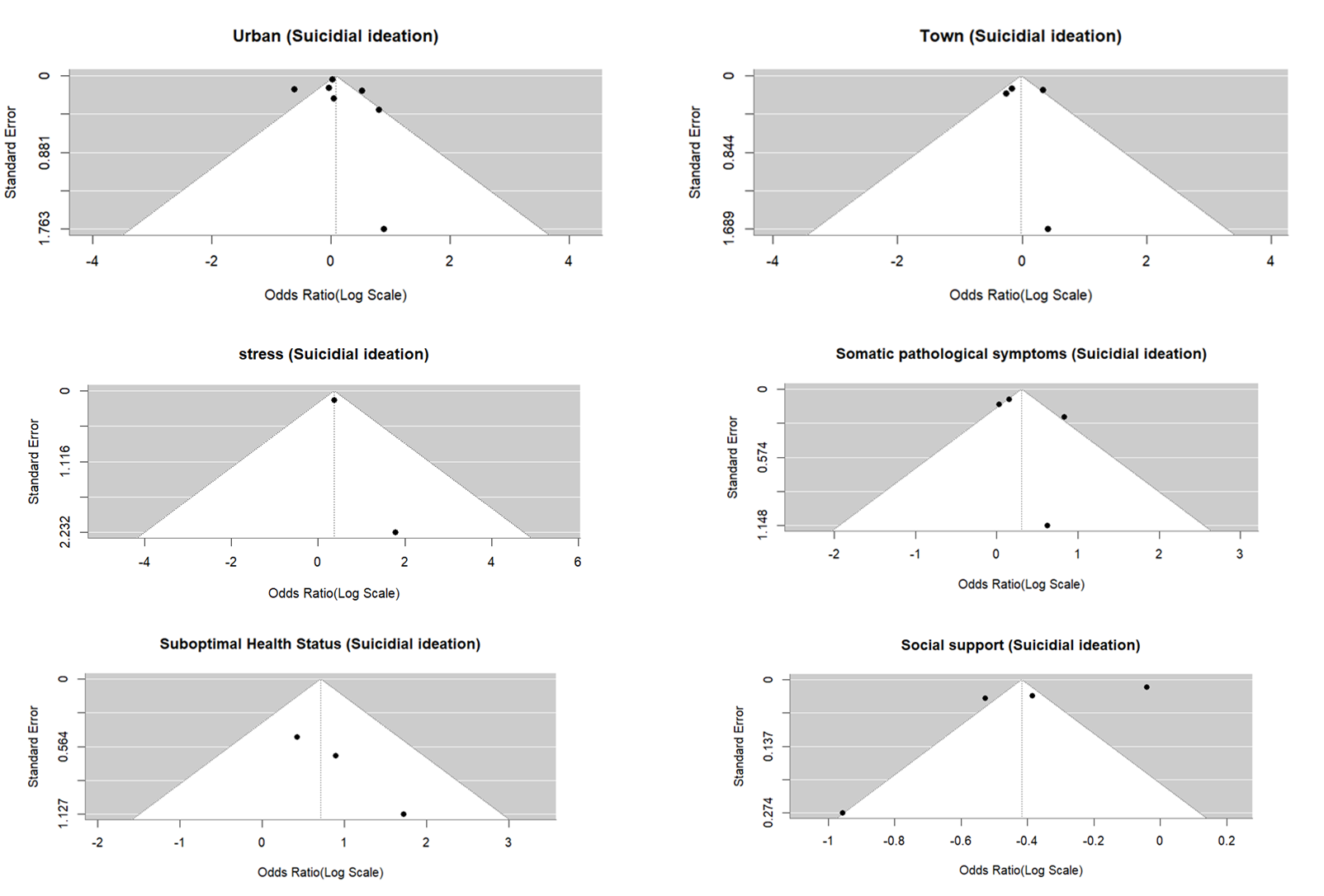


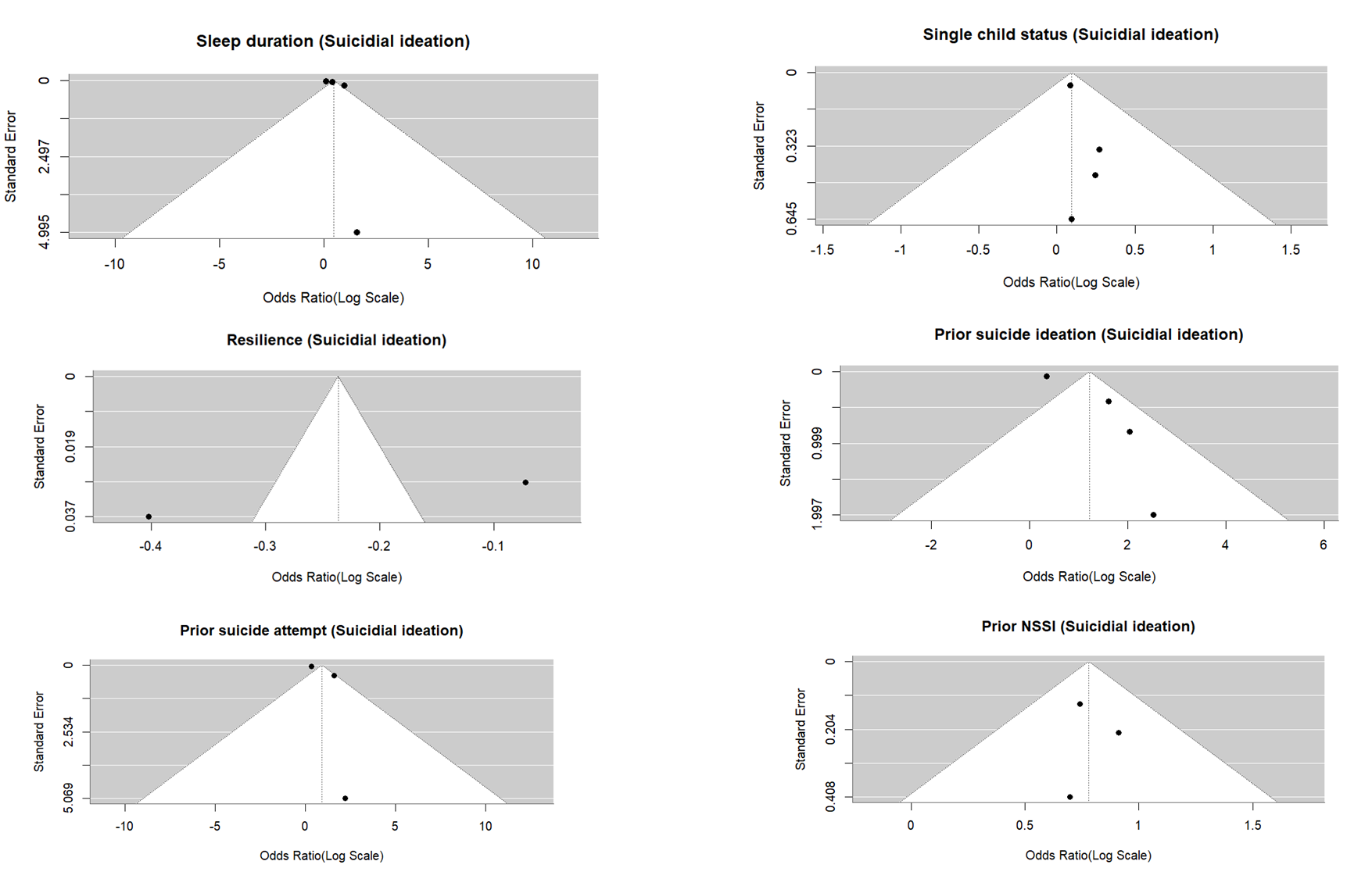


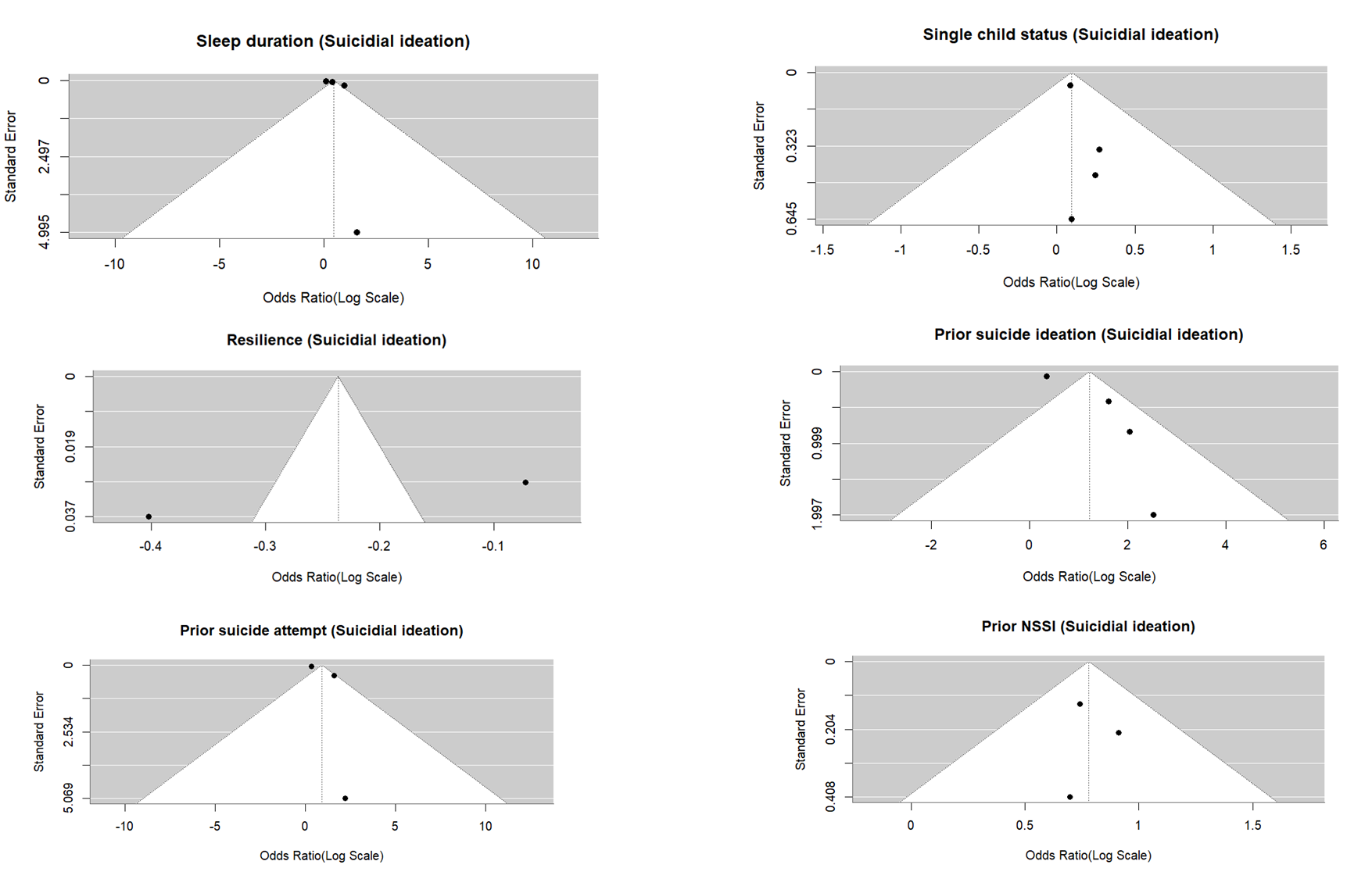


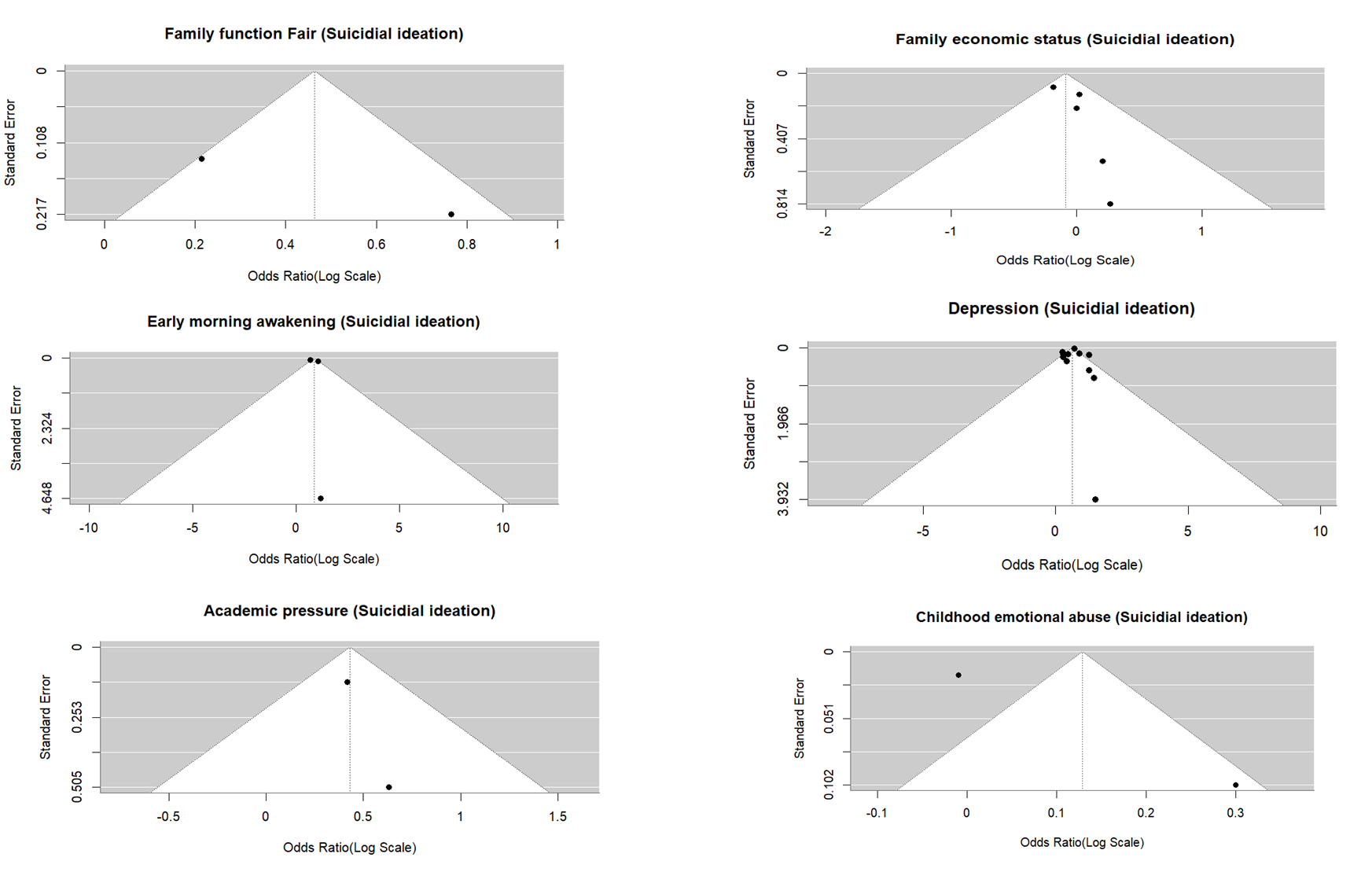


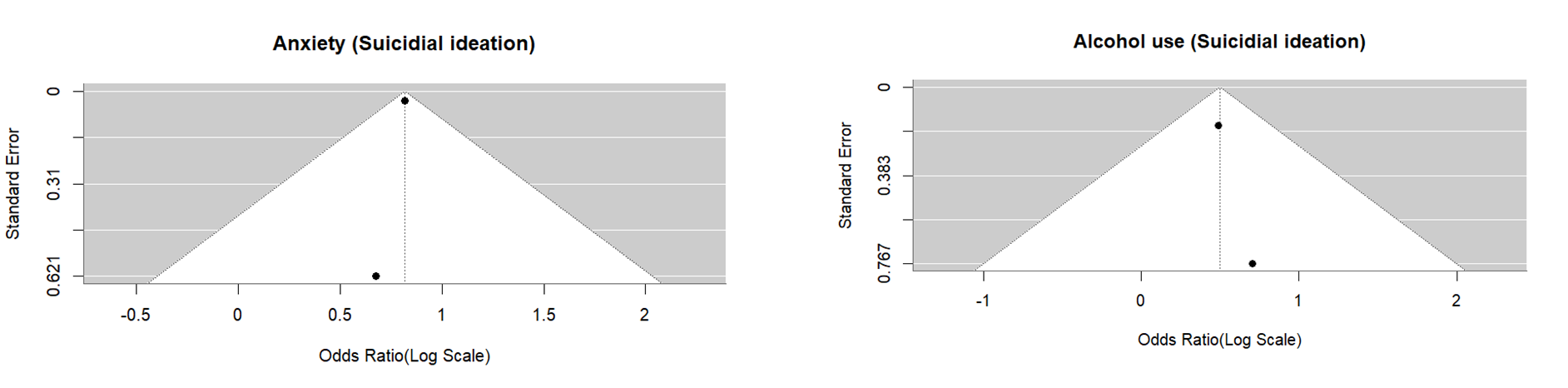

Supplement: Supplementary file 7 — Supplementary Material 7. [file 12889_2026_27430_MOESM7_ESM.docx]

# **Supplementary material 8** Funnel plot of suicide attempts


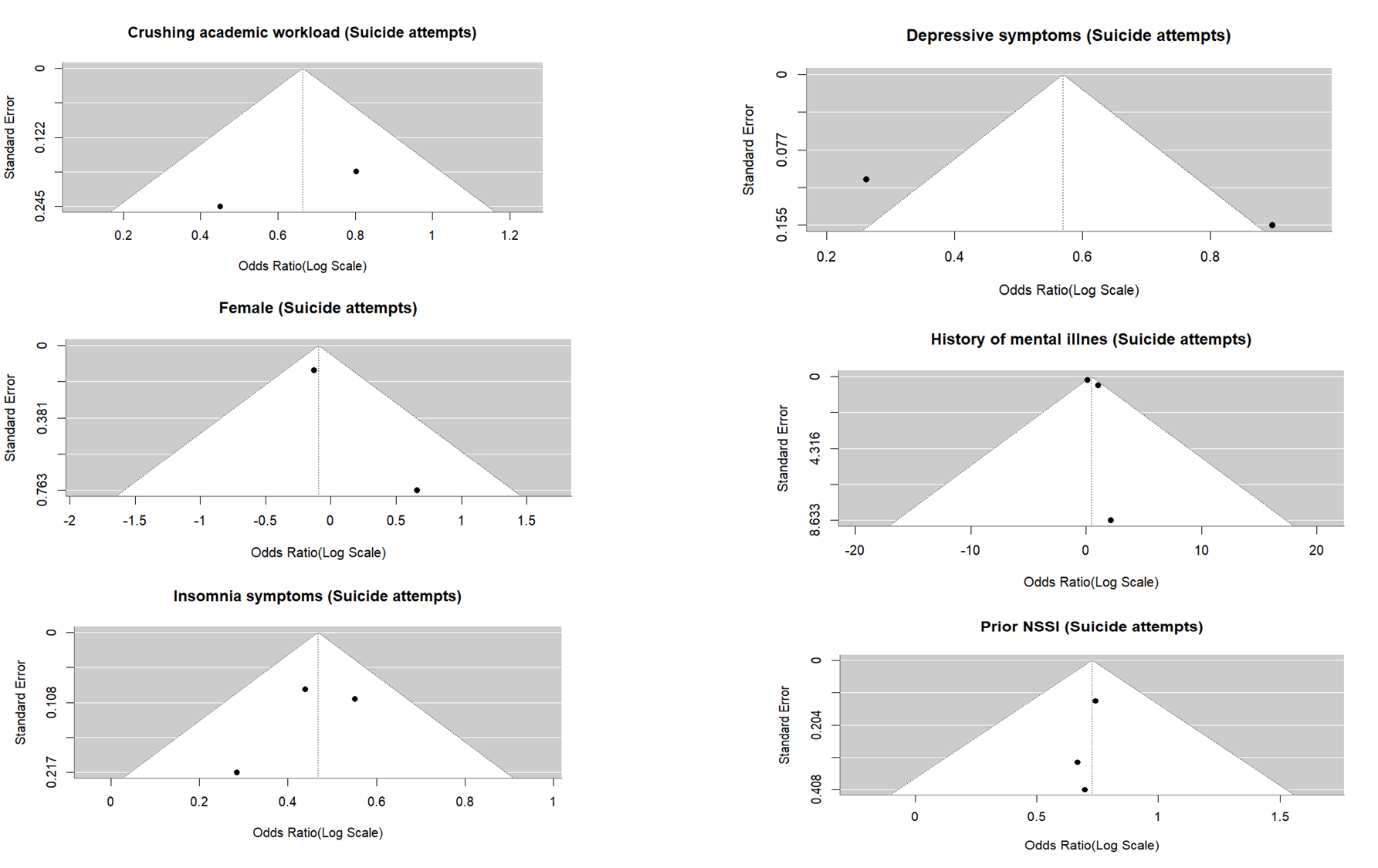


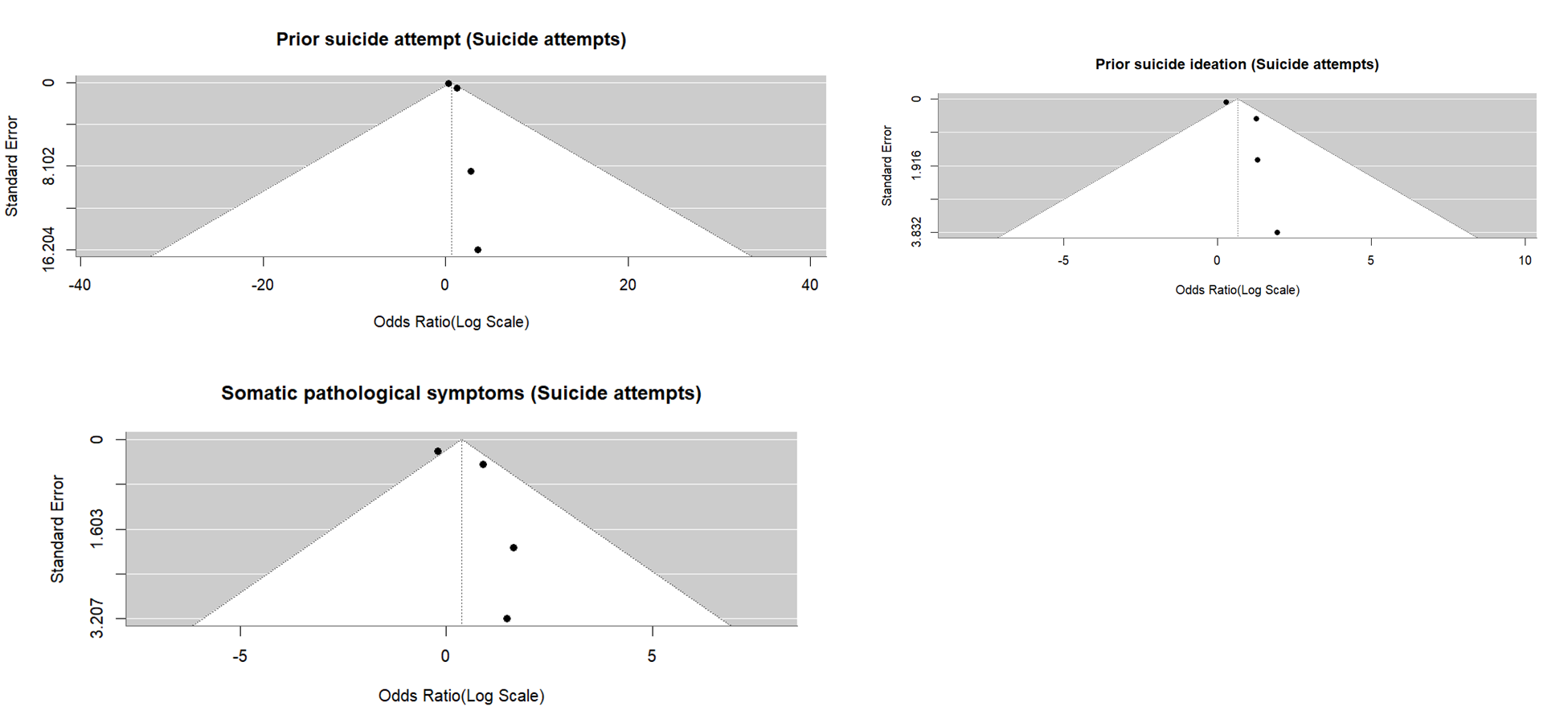

Supplement: Supplementary file 8 — Supplementary Material 8. [file 12889_2026_27430_MOESM8_ESM.docx]
