# Supplementary material for: Risk and protective factors for suicidal ideation and suicide attempts among Chinese university students: a systematic review and meta-analysis of longitudinal studies
Source: BMC Public Health. 2026 Apr 20;26:1787. doi: 10.1186/s12889-026-27430-0 (PMC13235143; doi:10.1186/s12889-026-27430-0)
Supplement: Supplementary file 9 — Supplementary Material 9. [file 12889_2026_27430_MOESM9_ESM.docx]

# **Supplementary material 9** Studies included in the meta-analysis

[1] Cao, H., Tao, F. B., Huang, L., Wan, Y. H., Sun, Y., & Su, P. Y., et al.(2012). Situation of common psychosomatic symptom in adolescent and its influence on 6 months later suicide[In Chinese]. Chin J Prev. Med. 46(3):202-208–80.

[2] Teng, S., Zhao, J. B., Zhang, X. Y., Zhao, J. B., Yang, X. L., & Chen, J.*(2018). Prevalence and influencing factors of suicidal ideation and suicide attempt among college students during the 2 years follow-up [In Chinese]. Chin J Sch Health. 39(8):1169–1172.

[3] Xing, C., Tu, C. Y., Tan, R. M., et al. (2010). Sub-health status and its implication on suicide among adolescent[In Chinese]. Chin J Sch Health. 31(12):1412–1414.

[4] Fu, W. X., Zhao, W. L., Yin, J. H., et al. (2022). The cross-lagged study between cyberbully and suicidal ideation among college students[In Chinese]. Chin J Sch Health. 43(3):399–402.

[5] Tu, C. Y., Huang, Z. H., Fang, Y. R., et al. (2011). Analysis of predictors of suicidal ideation among school adolescents[In Chinese]. Chin J Sch Health. 32(5):562–567.

[6] Wang, Y. R., Lu, D. F., Huang, S. H., Chen, C. N., & Lan, S. Q. (2024). Development trajectory and related factors of suicide ideation in college students [In Chinese]. *Modern Preventive Medicine*. 51(16):3010–3015.

[7] Yang, L. S. (2013). *The Prevalence of and Predictors for Suicidal Behaviors in the Following Year among College Students* [Master's thesis, Anhui Medical University]. China Doctoral Dissertations.

[8] Zhang, J. J., & Chen, H. (2021). A 4-year follow-up study on suicidal ideation of college students in one university [In Chinese]. Chin J Sch Health. 42(10):1524–1526.

[9] Zhang, P., Wang, P., Zhang, D., & Zhang, L. G. (2022). Effect of self-concept clarity and meaning in life on suicidal ideation in college fresh students[In Chinese]. Chin. Ment. Health J, 36(11), 975–980.

[10] Huang, S., Wang, D., Zhao, J., Chen, H., Ma, Z., Pan, Y., ... & Fan, F. (2022). Changes in suicidal ideation and related influential factors in college students during the COVID-19 lockdown in China. *Psychiatry Research*, *314*, 114653. <https://doi.org/10.1016/j.psychres.2022.114653>

[11] Li, Q., Chen, X., Zhu, Y., & Shi, X. (2024). Developmental pathways from insomnia to suicidality: A resilience perspective. *Journal of affective disorders*, *362*, 45-53. <https://doi.org/10.1016/j.jad.2024.06.104>

[12] Liu, L., Han, Y., Lian, Y., Wu, X., Qiao, Z., & Wang, W. (2023). Perceived discrimination and suicidal ideation among impoverished and nonimpoverished college students: Different mechanisms via social support, depressive symptoms, and nonsuicidal self‐injury. *Suicide and Life‐Threatening Behavior*, *53*(6), 910-921. [**https://doi.org/10.1111/sltb.12992**](https://doi.org/10.1111/sltb.12992)

[13] Liu, L., Wang, W., Lian, Y., Wu, X., Li, C., & Qiao, Z. (2024). Longitudinal impact of perfectionism on suicidal ideation among Chinese college students with perceived academic failure: The roles of rumination and depression. *Archives of suicide research*, *28*(3), 830-843. <https://doi.org/10.1080/13811118.2023.2237088>

[14] Ma, Z., Wang, D., Zhao, J., Zhu, Y., Zhang, Y., Chen, Z., ... & Fan, F. (2022). Longitudinal associations between multiple mental health problems and suicidal ideation among university students during the COVID-19 pandemic. *Journal of affective disorders*, *311*, 425-431. <https://doi.org/10.1016/j.jad.2023.07.108>

[15] Qiao, X., Shi, X., Chen, X., & Zhu, Y. (2023). Associations between insomnia symptom trajectories with depression and self-harm behaviors in Chinese college students before and during the COVID-19 pandemic: a five-wave longitudinal investigation. *Journal of affective disorders*, *339*, 877-886.

[16] Shi, X., Jiang, L., Chen, X., & Zhu, Y. (2022). Distinct trajectories of suicidal behaviors throughout the university stage and associated risk and protective factors: a large-scale prospective study. *Journal of affective disorders*, *319*, 407-415. <https://doi.org/10.1016/j.jad.2022.09.107>

[17] Shi, X., Zhu, Y., Wang, S., Wang, A., Chen, X., Li, Y., & Jiang, L. (2021). The prospective associations between different types of sleep disturbance and suicidal behavior in a large sample of Chinese college students. *Journal of affective disorders*, *279*, 380-387. <https://doi.org/10.1016/j.jad.2020.10.019>

[18] Sun, M., Wang, D., Jing, L., & Zhou, L. (2023). The predictive role of psychotic-like experiences in suicidal ideation among technical secondary school and college students during the COVID-19 pandemic. *BMC psychiatry*, *23*(1), 521. https://doi.org/10.1186/s12888-023-05025-y

[19] Wang, D., Ross, B., Zhou, X., Meng, D., Zhu, Z., Zhao, J., ... & Liu, X. (2021). Sleep disturbance predicts suicidal ideation during COVID-19 pandemic: a two-wave longitudinal survey. *Journal of psychiatric research*, *143*, 350-356. <https://doi.org/10.1016/j.jad.2021.11.033>

[20] Xu, H., Yang, X., Lai, X., Zhao, C., Tu, X., Ding, N., ... & Zhang, G. (2022). Longitudinal relationships among perceived stress, suicidal ideation and sleep quality in Chinese undergraduates: A cross-lagged model. *Journal of Affective Disorders*, *299*, 45-51.

[21] Yang, X., Wang, D., Liu, S., Liu, G., & Harrison, P. (2020). Stress and suicidal ideation: the role of state or trait anhedonia in a moderated mediation model. *Suicide and Life‐Threatening Behavior*, *50*(2), 502-514. [**https://doi.org/10.1111/sltb.12605**](https://doi.org/10.1111/sltb.12605)

[22] Zheng, D., Qin, Q., Peng, Y., Zhong, H., Huang, Y., Wang, H., Tan, Q., & Li, Y. (2024). Pre-COVID-19 short sleep duration and eveningness chronotype are associated with incident suicidal ideation during COVID-19 pandemic in medical students: A retrospective cohort study. *Frontiers in Public Health, 12*, Article 1406396. <https://doi.org/10.3389/fpubh.2024.1406396>.
